# Annotation of chromatin states in 66 complete mouse epigenomes during development

**DOI:** 10.1038/s42003-021-01756-4

**Published:** 2021-02-22

**Authors:** Arjan van der Velde, Kaili Fan, Junko Tsuji, Jill E. Moore, Michael J. Purcaro, Henry E. Pratt, Zhiping Weng

**Affiliations:** 1 Program in Bioinformatics and Integrative Biology, University of Massachusetts Medical School, Worcester, MA USA; 2 Bioinformatics Program, Boston University, Boston, MA 02215 USA

**Keywords:** Epigenetic memory, Genome informatics, Epigenomics, Gene regulation

## Abstract

The morphologically and functionally distinct cell types of a multicellular organism are maintained by their unique epigenomes and gene expression programs. Phase III of the ENCODE Project profiled 66 mouse epigenomes across twelve tissues at daily intervals from embryonic day 11.5 to birth. Applying the ChromHMM algorithm to these epigenomes, we annotated eighteen chromatin states with characteristics of promoters, enhancers, transcribed regions, repressed regions, and quiescent regions. Our integrative analyses delineate the tissue specificity and developmental trajectory of the loci in these chromatin states. Approximately 0.3% of each epigenome is assigned to a bivalent chromatin state, which harbors both active marks and the repressive mark H3K27me3. Highly evolutionarily conserved, these loci are enriched in silencers bound by polycomb repressive complex proteins, and the transcription start sites of their silenced target genes. This collection of chromatin state assignments provides a useful resource for studying mammalian development.

## Abstract (web summary)

van der Velde et al. present a resource for understanding changes in chromatin states during mouse prenatal development. The authors annotate ATAC-seq, whole-genome bisulfite sequencing, and ChIP-seq of data from Phase III of the ENCODE Project and find that bivalent regions are especially important for silencing lineage-specific genes, including master transcription factors.

## Introduction

Multicellular organisms maintain myriad cell types along distinct lineages to carry out cellular programs required for development and survival. These cell types have the same genome but different epigenomes, characterized by chromatin accessibility, histone modifications, and DNA methylation, which cooperate with trans-factors to regulate gene expression and downstream activities. Thus, systematic annotation of epigenomes is essential for understanding the genomic functions. Experimental techniques such as chromatin immunoprecipitation followed by sequencing (ChIP-seq)^1–3^, transposase accessible chromatin with sequencing (ATAC-seq)^4^, and whole-genome bisulfite sequencing (WGBS)^5^ enable genome-wide profiling of histone marks, chromatin accessibility, and DNA methylation, respectively. When several epigenetic marks have been profiled for a given cell type, computational algorithms such as ChromHMM^6^, Segway^7^, and IDEAS^8^ can integrate the results to classify genomic loci into distinct chromatin states predictive of their function.

**Fig. 1:**
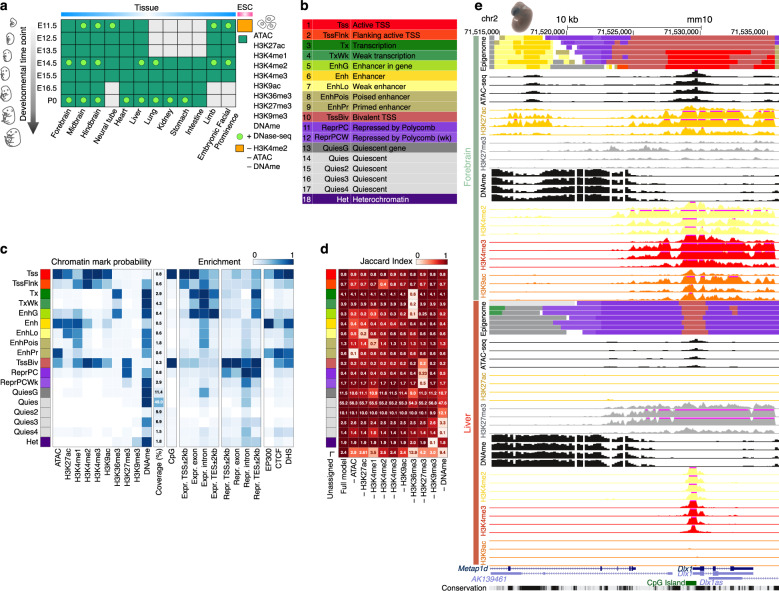
Overview of the 66 epigenomes and 18 chromatin states during mouse embryogenesis. **a** Twelve tissues at 4–7 developmental timepoints have ChIP-seq data for eight histone marks (green boxes), ATAC-seq data, and DNA methylation (DNAme) data, totaling 66 complete epigenomes. Twenty-one of these epigenomes also have DNase-seq data (green dots). Embryonic stem cells (orange box) have ChIP-seq data for seven histone marks, and are missing H3K4me2, ATAC-seq, and DNAme. **b** Eighteen chromatin states are defined by ChromHMM across the 66 complete epigenomes. **c** Histone-mark probabilities, genome coverage (averaged over 66 epigenomes, posterior probability > 0.5), and overlapping genomic features including gene expression, regulatory features (EP300 binding, CTCF binding, and DNase I hypersensitive sites), and distances to the TSSs of expressed and repressed genes are shown for each chromatin state. The enrichments for the categories are the averaged values across tissues and timepoints. **d** Jaccard similarities between the partial epigenomes with each mark omitted and the ten-mark E13.5 midbrain epigenome. **e** The *Dlx1* locus is displayed with chromatin states (color-coded as in **b**) in the forebrain and the liver for all seven timepoints. Also shown are the signals of several histone marks (scale: 0–50) that differ between forebrain and liver (for E11.5, E13.5, E15.5, and P0 only, due to space constraints), along with ATAC and DNA methylation signals. A transgenic mouse embryo is shown on top of the enhancer region, indicating the forebrain-specific activity of this enhancer. A CpG island that overlaps with the bivalent region at the TSS of *Dlx1* is shown at the bottom of the panel.

Coordinated efforts by the ENCODE consortium and the Roadmap Epigenomics consortium provided tremendous insights into gene regulation in a diverse array of human cell and tissue types^9,10^. The mouseENCODE project furthered understanding of mouse tissue and cell types in adults and at one developmental timepoint (embryonic day 14.5; E14.5)^11^. ENCODE Phase III generated 66 complete mouse epigenomes across 12 fetal tissues at four to seven developmental timepoints, each investigated with ten assays^12^: ATAC-seq^13^, WGBS^14^, and ChIP-seq of eight histone marks^13^. The histone marks included histone 3 lysine 4 trimethylation (H3K4me3) and histone 3 lysine 9 acetylation (H3K9ac), enriched at promoters and present at enhancers^1,15–17^; H3K27ac, H3K4me1, and H3K4me2, enriched at enhancers^1,15,17,18^; H3K36me3, enriched within bodies of actively transcribed genes^19^; H3K27me3, catalyzed by and guiding the polycomb repressive complexes (PRC) of proteins to repress gene expression^20^; and H3K9me3, enriched in heterochromatin to silence repeats and gene clusters^19^. All 66 epigenomes were accompanied by transcriptome sequencing (RNA-seq)^21^ and DNase-seq, another technique for measuring chromatin accessibility^22^ (Fig. 1a and Supplementary Data 1). This collection represents the most complete epigenetic data set of fetal mouse tissues, ideal for characterizing the epigenomic landscape of mammalian development.

We applied ChromHMM^6^ to these 66 mouse epigenomes, and defined 18 chromatin states (Fig. 1b). Most of these recapitulated the 15 human chromatin states defined by the Roadmap Epigenomics Consortium on five-mark human epigenomes^10^ and the 15 mouse chromatin states defined by the Ren lab using only the eight histone modifications^13^. Some of the states in our ten-mark, 18-state model corresponded to refinements of previously defined enhancer, bivalent, and quiescent states.

We further investigated one chromatin state in detail—TssBiv, a bivalent state enriched in transcription start sites (TSS) harboring both active marks (H3K4me3, H3K4me2, H3K4me1, and H3K9ac) and the repressive mark H3K27me3. Genes with bivalent TSSs were first identified in embryonic stem cells and thought to be poised for activation or repression in response to developmental or environmental cues^23^. Subsequently, bivalent regions have been extensively studied in developmental and adult tissue and cell types^24–30^ (for details, see recent reviews^31,32^). The ENCODE epigenomes provide the opportunity to analyze bivalent domains across tissues and developmental timepoints systematically. We find that TssBiv loci are substantially more evolutionarily conserved than loci in the other 17 chromatin states. Each fetal tissue harbors ~3000 bivalent genes; many are transcription factors (TFs) repressed in the given tissue and differentially expressed among the others. Comparison with recently defined silencers bound by the PRC2 proteins^33^ revealed that both silencers and the TSSs of their silenced genes are highly enriched in the bivalent regions. Thus, bivalent regions support an evolutionarily conserved silencing mechanism for lineage-specific genes, particularly master TFs controlling tissue development. Our comprehensive annotation of chromatin states provides a resource for studying mammalian development.

## Results

### Chromatin states are defined using ATAC-seq, WGBS, and the ChIP-seq data of eight histone marks

The 66 fetal mouse epigenomes from the C57BL/6 strain, all complete with ten chromatin marks, represent a comprehensive collection for chromatin state assignment (Fig. 1a). We used ChromHMM to learn 18 states jointly from this data set (Fig. 1b, c). ChromHMM divides the genome into non-overlapping 200 base-pair (bp) bins, which it assigns to one of the 18 chromatin states in each biosample. We named our chromatin states to maximize consistency with earlier ChromHMM publications^6,10,34^; two are proximal to active TSSs (Tss and TssFlnk, on average occupying 1.4% of the mouse genome); two states associate with actively transcribed genes (Tx and TxWk, 7.2%); five states are enhancer-related (Enh, EnhLo, EnhPois, EnhPr, and EnhG; 3.9%); one bivalent state often falls near inactive TSSs (TssBiv, 0.3%); three states are repressive (ReprPC and ReprPCWk enriched in H3K27me3, 3.7%; and Het in H3K9me3, 1.8%); and five states are quiescent (QuiesG, Quies, Quies2, Quies3, and Quies4; 78.7%). The remaining 3% of the genome could not be confidently assigned to any one state (denoted “unassigned”; posterior probability less than 0.5).

State assignments are supported by comparison with gene expression and epigenomic data available for a subset of biosamples (Supplementary Data 1). Although both the active-TSS (Tss) and the bivalent TSS states (TssBiv) are highly enriched in CpG islands, Tss (along with the TSS-flanking state TssFlnk) is only enriched in the TSSs of expressed genes (determined using RNA-seq data in the corresponding biosample). In contrast, TssBiv is only enriched at TSSs of repressed genes (Fig. 1c). The transcription-related states (Tx and TxWk) are enriched in exons and introns of expressed genes but not repressed genes (Fig. 1c). Enh (high-signal enhancer) is the state most enriched in ChIP-signal for EP300, a histone acetyltransferase that preferentially binds active enhancers^35,36^ (Fig. 1c). The relative enrichment of the 18 states for ATAC-seq signal is consistent with enrichment in DNase hypersensitive sites (DHS) determined using DNase-seq data in the corresponding biosample (Fig. 1c).

### Contributions of the chromatin marks to the assignments of chromatin states

To assess the contribution made by each of the eight histone marks, ATAC, and DNA methylation, we asked how accurately the ten-mark model would be able to annotate a new epigenome missing data for one of the marks. We addressed this question using the midbrain E13.5 epigenome by removing the data for each mark individually and computing the Jaccard similarity index between the chromatin state assignments of all genomic bins (each 200-bp long, which is the resolution of ChromHMM) using the data for the remaining nine marks. In general, when a mark is removed, the states most severely affected were among those states most enriched in this mark in the ten-mark model (compare Fig. 1d and the chromatin-mark probabilities in Fig. 1c). However, the converse is not necessarily true, reflecting the redundancy between the marks. For example, the removal of H3K27ac affects the low-signal enhancer state (EnhLo) although the high-signal enhancer (Enh) state is even more enriched in H3K27ac than EnhLo (Fig. 1c–d). H3K4me3 and H3K9ac, when removed individually, did not have a major impact on any of the states although promoter states are enriched in H3K4me3 and both promoter and enhancer states are enriched in H3K9ac (Fig. 1c–d), indicating that the information contained by each of these two marks is already accounted for by the other nine marks. On the other hand, H3K36me3, H3K27me3, and H3K9me3 each brings non-redundant information to the ten-mark model, as all the states enriched in each of these marks were affected when the mark was removed (Fig. 1c–d).

### Chromatin states are conserved between human and mouse

The Roadmap Epigenomics Consortium previously defined 15 human chromatin states using five histone marks in 127 human biosamples^10^. To investigate the conservation of chromatin state types between human and mouse, we built a 15-state model using the same set of five histone marks in the 66 mouse fetal biosamples (Supplementary Fig. 1a). Visual comparison of the chromatin states in our five-mark 15-state mouse model and those in the five-mark 15-state human model by the Roadmap Epigenomics Consortium revealed that 13 states had similar emission probabilities. These 13 states, including the promoter, enhancer, transcribed, repressed, and bivalent states, were enriched in at least one of the five histone marks. The remaining two human states—the weak transcription state TxWk and the weak repressed polycomb state ReprPCWk (11.6% and 8.3% of the human genome)—had low signals for all five marks^10^. Instead, we identified in mouse a QuiesG state with low signals for all marks and a minor state TxWk marked by low levels of H3K36me3 and H3K27me3 (25.17% and 0.13% of the genome). In summary, our results indicate that the chromatin states are highly conserved between human and mouse, and ChromHMM is able to identify these states reliably.

### Addition of histone marks, chromatin accessibility, and DNA methylation further clarifies enhancer, bivalent, and quiescent states

We built another 15-state mouse model using all eight available histone marks (Supplementary Fig. 1a), similar to the model in another ENCODE3 companion paper^13^ (see Methods). As additional marks are incorporated, state assignments differ predominantly for the enhancer, bivalent, and quiescent states (Supplementary Fig. 1). The five-mark model specified one enhancer state (Enh; 3.7% of the mouse genome) with high H3K4me1 (Supplementary Fig. 1a). Genomic regions in this state were assigned to five distinct enhancer states in the eight-mark model reflecting different levels of the additional enhancer marks (H3K4me2, H3K9ac, and H3K27ac). Among these five states, the high-signal enhancer state Enh, which showed high levels for all of four enhancer marks, occupied only 0.2% of the genome (Supplementary Fig. 1a, d). The Enh state defined by the ten-mark model further showed high chromatin accessibility (ATAC signal) and low DNA methylation, occupying 0.64% of the genome (Supplementary Fig. 1a, d). The ten-mark model defined three additional enhancer states, with two of them (EnhLo and EnhPois) being regroupings of the genomic regions assigned to the enhancer states in the eight-mark model, and other state (EnhPr) corresponding to a subset of the regions assigned one of the enhancer states by the eight-mark model, showing high chromatin accessibility but low levels of enhancer marks (Supplementary Fig. 1d). Thus, the additional marks led to refined definitions of enhancer states.

One example of a tissue-specific enhancer is located inside the housekeeping gene *Metap1d* (methionyl aminopeptidase Type 1D) and 10 kb upstream of the *Dlx1* gene, which encodes a brain-specific homeobox TF. *Dlx1* is highly expressed in the forebrain (~200 transcripts per million or TPM) but not expressed in most other tissues including the liver. This enhancer region is annotated as a high-signal Enh in the forebrain, showing high ATAC and H3K27ac signals and low DNA methylation. It is annotated as a quiescent gene (QuiesG) in the liver owing to its low ATAC and histone-mark signals and high DNA methylation (Fig. 1e). A VISTA enhancer (accession: hs553) overlapping this region is active in the forebrain and cranial nerve of mouse embryos^37^.

The five-mark model annotated three bivalent states with high levels of the active marks H3K4me1 and H3K4me3, as well as the repressive mark H3K27me3; however, the eight-mark and ten-mark models only annotated one bivalent state, which additionally showed high levels of H3K4me2, H3K9ac, and ATAC and low DNA methylation (Supplementary Fig. 1a). Roughly the same set of genomic regions were assigned to these bivalent states across the three models, suggesting that some of the states that would capture distinctions among bivalent regions are now used to capture distinctions among other regions enabled by the additional marks, e.g., additional enhancer states as described above (Supplementary Fig. 1e).

The five-mark and eight-mark models annotated one quiescent state (Quies) having very low signals for all available histone marks. The ten-mark model defined three additional quiescent states besides Quies, which exhibit low levels of histone marks and ATAC but varying levels of DNA methylation (Supplementary Fig. 1a). The quiescent states in the three models cover roughly the same genomic regions (Supplementary Fig. 1b). Among the 18 states of the ten-mark model, Quies2 has the highest percentage of genomic bins without any CpG dinucleotides (50%; Supplementary Fig. 2a). For no-CpG bins, DNA methylation was labeled as “missing data” (see Methods); nonetheless, Quies2 bins with one or more CpGs also show low DNA methylation, whereas the other three quiescent states show higher DNA methylation levels regardless of CpG count (Supplementary Fig. 2b–e). In contrast, the Tss state shows low DNA methylation anticorrelated with CpG count (Supplementary Fig. 2f), consistent with previous observations for promoters^38^.

### Some regions in the quiescent states may be in low-signal H3K27me3 and H3K9me3 domains

We define two types of repressive states: ReprPC and ReprPCWk, the two states highly enriched in H3K27me3, jointly occupy 3.7% of the mouse genome, and Het, the state highly enriched in H3K9me3, occupies 1.8% of the mouse genome (Fig. 1c). However, Zaret and colleagues reported much larger genomic footprints for H3K27me3 domains (10.6% of the human genome) and H3K9me3 domains (19.6% of the human genome) in BJ fibroblasts^39^. Thus, we directly examined the 15-state five-mark model by the Roadmap Epigenomics Consortium on a similar human cell line, IMR90 lung fibroblast, which assigned 4.4% and 13.8% of the human genome to ReprPC and ReprPCWk, overlapping 24.6% and 43.5% of Zaret’s H3K27me3 domains, respectively, and 8.5% of the genome to Het, occupying 29.8% of Zaret’s H3K9me3 domains. Meanwhile, 17% of Zaret’s H3K27me3 domains and 60% of Zaret’s H3K9me3 domains were in the Quies state although the ReprPC and ReprPCwk states were the most enriched in H3K27me3 domains and the Het state was the most enriched in H3K9me3 domains. Our 18-state ten-mark mouse model defines five quiescent states (Quies, Quies2, Quies3, Quies4, QuiesG) collectively occupying 80.5% of the mouse genome. These states show closed chromatin, very low levels of histone marks, and varying levels of DNA methylation. Except for Quies2, the other four quiescent states show low levels of H3K27me3 and H3K9me3 (Fig. 1c), the two repressive histone marks, and could encompass some of the H3K27me3 and H3K9me3 domains.

### Variation of state assignments across tissues and developmental timepoints

**Fig. 2:**
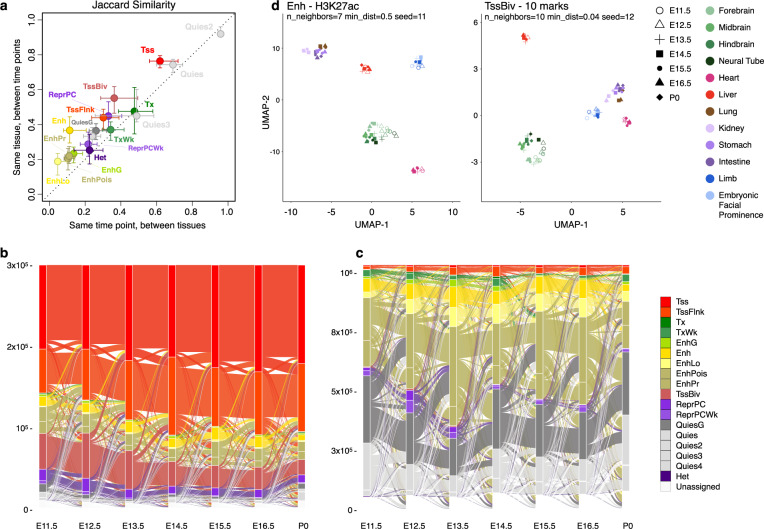
Variations of the chromatin states across tissues and their transitions along the developmental trajectory. **a** Jaccard similarity between different timepoints in the same tissue (*y* axis) versus the similarity between different tissues at the same timepoint (*x* axis). Error bars indicate the range between the first and third quartiles. **b** Transitions between chromatin states along midbrain developmental timepoints. For clarity, only the genomic bins assigned TSS-related states (Tss, TssFlnk, and TssBiv) at one or more timepoints are included. **c** Same as **b** but for genomic bins assigned enhancer-related states (Enh, EnhLo, EnhPois, and EnhPr) at one or more developmental timepoints. **d** Visualization of the 66 epigenomes in two dimensions using the UMAP technique. (Left) UMAP was given the H3K27ac signals in the Enh genomic bins across the 66 epigenomes. There were 735,048 such genomic bins, which were assigned Enh in one or more epigenomes. (Right) UMAP was given the signals of all 10 marks in the TssBiv genomic bins across the 66 epigenomes. There were 156,752 such genomic bins, which were assigned TssBiv in one or more epigenomes.

We assessed variability among state assignments across the 66 mouse epigenomes with the Jaccard similarity index. Enhancer states and the repressive Het state exhibited the greatest variability among tissues or across timepoints, whereas the quiescent (especially Quies2), promoter, and transcription states showed the least variability (Fig. 2a). Moreover, all states were more similar across timepoints in the same tissue than across tissues at the same timepoint (Fig. 2a), consistent with the notion that the epigenome is inherited within cell lineages. Temporal chromatin state transitions for each tissue occurred mostly between related states, e.g., among the promoter states (Tss, TssFlnk, and TssBiv) or among the enhancer states (Enh, EnhLo, EnhPois, and EnhPr) (Fig. 2b, c).

To investigate whether variations in chromatin states could recapitulate the developmental trajectory, we applied the UMAP dimension-reduction technique^40^ to the 66 epigenomes. H3K27ac signal within Enh regions (5.4% of the genome) cleanly segregated the biosamples by tissue (Fig. 2d, left panel), as did signals for the ten chromatin marks at bivalent regions (TssBiv, 1.2% of the genome; Fig. 2d, right panel). The two organs that differentiate early, the heart (with a mesoderm origin) and the liver (endoderm), formed two separate clusters. Tissues with similar developmental origins were positioned near each other, with the four brain regions (ectoderm), the lung and the digestive tract (endoderm), and the limb and facial prominence (with cells from both endoderm and ectoderm origins) forming three clusters (Fig. 2d). The kidney (mesoderm) biosamples positioned near the endoderm biosamples. The liver does not cluster with the other endoderm organs likely because it differentiates much earlier, becoming a functional organ of hematopoiesis from E11.5 to E16.5^41^ and undergoing global demethylation during this process^14^. In contrast, the lung, stomach, and intestines do not need to be functionally differentiated until birth. The limb and facial prominence tissues clustered together, reflecting parallels in the development of these two organs^42,43^. Furthermore, within each tissue, the earlier timepoints (open symbols in Fig. 2d) were segregated from later timepoints (filled symbols). Thus, the epigenomic landscapes captured by chromatin states Enh and TssBiv can accurately recapitulate the tissue lineages during fetal development.

### Genome regions transit among TssBiv, Tss, and ReprPC states

Bivalent promoters in embryonic stem cells are “resolved” to become monovalent upon differentiation, with activated genes losing H3K27me3 and repressed genes losing H3K4me3^44^. Similar results have been reported for cells committed to various lineages^26,27,29^. Consistent with earlier results in both embryonic stem cells and differentiated cell lineages^26,27,29,30,44,45^, we found that over developmental time, regions assigned to the bivalent promoter state (TssBiv), which has both active marks and the repressive H3K27me3 mark (Fig. 1c), can either lose repressive H3K27me3 and become active TSSs (Tss) or lose the active marks and transition into the repressive polycomb (ReprPC) state (Supplementary Fig. 3a). For example, the promoter of the *Dlx1* gene is annotated as Tss in the forebrain, where it is highly expressed, and TssBiv in the liver, where it is not expressed and surrounded by ReprPC regions (Fig. 1e).

Roughly 0.3% of any particular epigenome is assigned to the TssBiv state; cumulatively 1.2% of the genome is assigned to TssBiv across all tissues and timepoints. TssBiv is less than half as prevalent as Tss and ReprPC, which constitute 0.8% and 0.8% of each epigenome and 2.2% and 5.5% of the genome overall, respectively. Almost all stretches of TssBiv genomic bins are flanked by ReprPC genomic bins. Among the genomic bins that are assigned TssBiv in any of the epigenomes, 64.7% are assigned ReprPC in at least one epigenome and 68.1% are assigned Tss in at least one epigenome (Supplementary Fig. 3b), indicating that a particular region is TssBiv in some tissue but becomes monovalent (Tss or ReprPC) in other tissues. Intriguingly, the overall fraction of TssBiv genomic bins decreased over the course of the development in all five tissues with seven timepoints, although due to the small number of timepoints this was statistically significant only in the three brain tissues (Supplementary Fig. 3c). This suggests that the resolution of TssBiv regions into a monovalent state is important for development, especially in the brain.

### Bivalent genes are involved in fundamental biological processes

We identified 14,558 bivalent regions, defined as stretches of TssBiv genomic bins surrounded by repressive chromatin states in any of the 66 biosamples (see Methods). These bivalent regions overlapped 14,729 GENCODE-annotated TSSs (Supplementary Data 2), belonging to 6800 genes (Supplementary Data 3). There were 1077 genes that were bivalent in all 12 tissues (i.e., having at least one bivalent TSS at one or more timepoints of every tissue), and these genes were highly enriched in Gene Ontology (GO) terms related to embryonic development of myriad organs and systems, regulation of fundamental cellular processes, and modulation of cell–cell communications (Supplementary Fig. 4a and Supplementary Data 4a, b).

The liver had 5482 bivalent genes (i.e., having at least one bivalent TSS at one or more timepoints), 74% more than the other 11 tissues on average, and 1291 of these 5482 genes were not bivalent in the other 11 tissues. This is because the liver genome undergoes global CpG demethylation from E11.5 to E16.5, precisely coincident with the onset of fetal liver haematopoiesis, showing large swaths of the genome in partially methylated domains that are enriched in H3K9me3 and H3K27me3 but depleted of H3K27ac^14^. This phenomenon is likely caused by the failure of mCG maintenance in rapidly dividing cells during haematopoiesis^14^. GO analysis on the 1291 liver-only bivalent genes revealed terms that were involved in the development of a wide variety of organs other than the liver, such as heart, kidney, smooth muscle, brain, and cytoskeleton (Supplementary Fig. 4b and Supplementary Data 4c, d). We observed similar results for bivalent genes specific to other tissues. Thus, the bivalent genes in each fetal tissue reflect the regulatory pathways that are unused by the developmental program of that specific tissue. Our results are consistent with earlier findings on bivalent genes in other cell and tissue types^23,26–30,45,46^.

### Bivalent genes exhibit repressed transcription

**Fig. 3:**
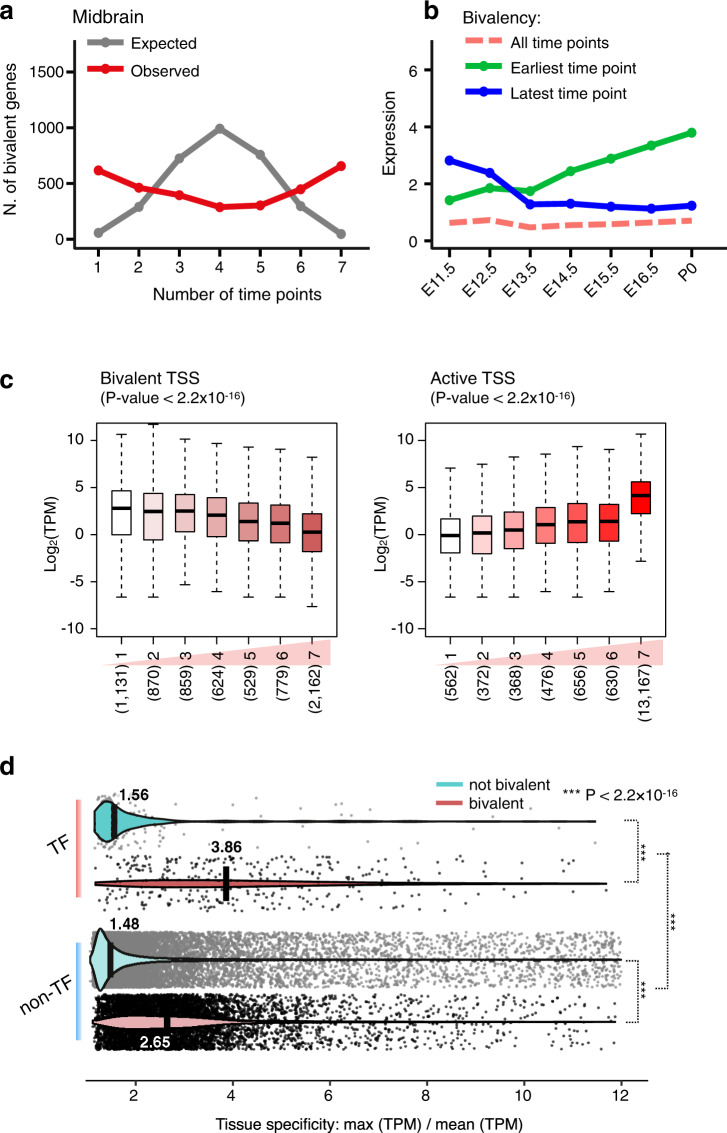
Count and expression of bivalent genes along developmental timepoints. **a** The number of bivalent genes at 1–7 timepoints in the midbrain. Observed and expected numbers of genes are in red and in gray, respectively. **b** Median expression levels of three groups of genes: (green) bivalent at the earliest timepoint but not at the last timepoint, (blue) bivalent at the last timepoint but not at the first timepoint, and (pink) bivalent at all timepoints. **c** Distribution of gene expression, with genes grouped by the total number of timepoints at which their TSSs are in the bivalent state TssBiv (left) or in the active state Tss (right) in the forebrain. The total number of genes in each group is shown below each boxplot in parentheses. For all boxplots, whiskers show 95% confidence intervals, boxes represent the first and third quartiles, the vertical midline is the median, and outliers are omitted. There is a negative correlation between expression and the duration of the bivalent state and a positive correlation between expression and the duration of the active state (*P* values < 2.2 × 10^−16^). **d** Violin plots show the distributions of tissue specificity scores for bivalent and non-bivalent genes that encode transcription factors (TFs) and non-TFs. Medians are shown in black bars with values indicated. *P* values are shown for three comparisons as indicated.

Earlier studies of bivalent genes revealed that they are poised or repressed for transcription^23,26–30,45,46^. We further analyzed the expression of the 25,215 genes that were expressed (≥1 TPM) in at least one of the 66 biosamples, among which 6324 were among our list of bivalent genes (see Methods). We found that the bivalent genes in a tissue had lower expression levels than non-bivalent genes according to RNA-seq data in the same tissue. Across the 66 biosamples, the expression levels of bivalent genes were 5.2 ± 1.7 TPM, much lower than the expression levels of non-bivalent genes (39.8 ± 2.1 TPM; Wilcoxon rank-sum test *P* value < 2.2 × 10^−16^). Furthermore, the genes that were not bivalent in any of the timepoints of a tissue were expressed 7.79-fold higher (Wilcoxon rank-sum test *P* values ≤ 2.2 × 10^−16^) than the genes that were bivalent at all timepoints of the tissue (Supplementary Fig. 5). In a particular tissue, genes that were bivalent at different timepoints were largely consistent (forebrain in Fig. 3a; all tissues in Supplementary Fig. 6). For example, 1830 genes were bivalent at all seven timepoints of the liver; only 439 such genes would be expected if the timepoints were independent of one another (*P* value < 2.2 × 10^−16^; Binomial test). Genes bivalent at the earliest timepoint but not the latest timepoint were expressed at significantly lower levels earlier in development; likewise, genes bivalent at the latest timepoint but not at the earliest timepoint were expressed at lower levels later in development (midbrain in Fig. 3b; all tissues in Supplementary Fig. 7). Both of these two sets of genes were expressed at significantly higher levels than genes bivalent at all timepoints in the same tissue (Fig. 3b, Supplementary Fig. 7). Overall, the average expression level of a TSS across the timepoints in a tissue is anticorrelated with the number of timepoints at which the TSS is in a genomic bin assigned to the TssBiv chromatin state; in sharp contrast, a positive correlation is observed between expression and the duration the TSS is in a genomic bin assigned to the Tss chromatin state (Fig. 3c; Supplementary Fig. 8). Thus, the expression of bivalent genes is repressed in a tissue- and timepoint-specific manner.

### Bivalent genes are highly enriched in tissue-specific TFs

We compared the 6797 bivalent genes (6324 expressed in at least one of the 66 biosamples) with a curated list of 552 TFs with known DNA binding motifs in both mouse and human^47^, of which 535 were expressed in at least one of the 66 biosamples. A majority of the 535 TFs (338, 63.2%) were among the 6324 bivalent genes (Chi-square *P* value < 2.2 × 10^−16^), consistent with earlier findings in embryonic stem cells^23^. Bivalent genes, both TF and non-TF, were significantly more tissue-specific than non-bivalent genes (2.47-fold and 1.79-fold higher median tissue specificity for TFs and non-TFs, respectively, Wilcoxon rank-sum test *P* values < 2.2 × 10^−16^; Fig. 3d).

Consistent with earlier findings in embryonic stem cells^48,49^, a majority of the bivalent TSSs in our mouse fetal biosamples (mean = 62.5% across the 66 biosamples) overlapped CpG islands; in contrast, only a minority of non-bivalent TSSs did (mean = 29.8%; Chi-square *P* values in all 66 biosamples < 2.2 × 10^−16^). The enrichment holds for both TF genes (mean = 64.4% for bivalent TSSs vs. 43.5% for non-bivalent TSSs; *P* values < 2.2 × 10^−16^) and non-TF genes (62.3% vs. 29.5%, *P* value < 2.2 × 10^−16^). CpG promoters are known to be less tissue-specific than non-CpG promoters^38^, which seems to conflict with our above finding that bivalent genes are significantly more tissue-specific than non-bivalent genes (Fig. 3d). To investigate the apparent contradiction, we separated bivalent and non-bivalent TSSs into CpG and non-CpG sub-groups. Indeed, each CpG subgroup is significantly less tissue-specific than the non-CpG subgroup with the same valency; however, the bivalent CpG subgroup is significantly more tissue-specific than the non-bivalent CpG group (Supplementary Fig. 9). Specifically, bivalent CpG TFs (*N* = 314) are significantly more tissue-specific than non-bivalent CpG TFs (*N* = 162; Wilcoxon rank-sum test *P* value < 2.2 × 10^−16^).

**Fig. 4:**
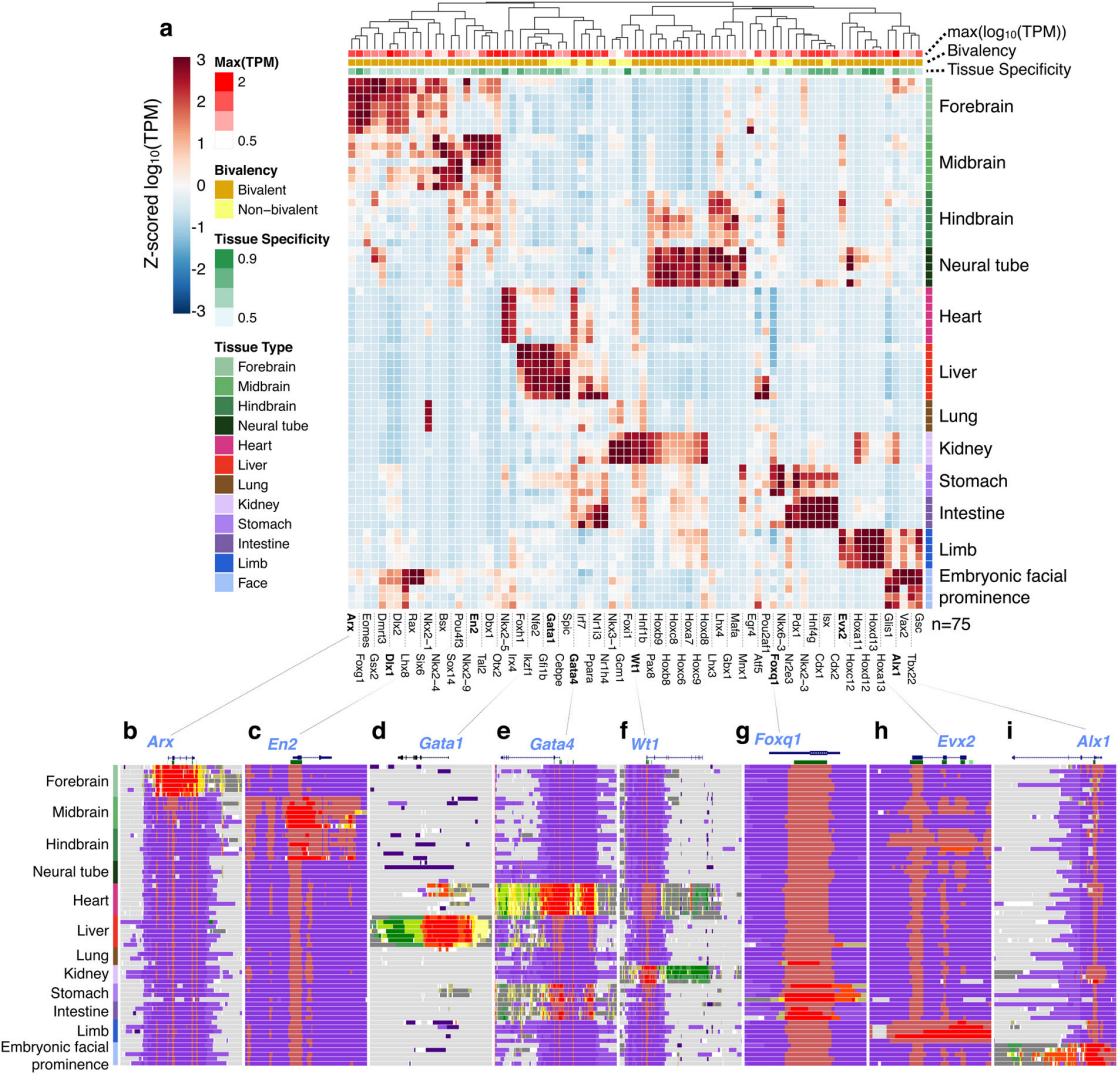
Expression profiles and chromatin states for the transcription factors with the highest tissue specificity scores. **a** Hierarchical clustering of expression profiles for the TFs with tissue specificity scores >6, with 75 TFs in total. Rows on the top show the maximal expression level across all biosamples (intensities of red), bivalency status (brown for 62 bivalent TFs, and yellow for 13 non-bivalent TFs), and tissue specificity score (intensities of green). **b**–**i** Example TFs and the chromatin state assignments near their loci. Among these, Gata1 (**d**) is a non-bivalent TF and the rest are bivalent TFs: Arx (**b**), En2 (**c**), Gata4 (**e**), Wt1 (**f**), Foxq1 (**g**), Evx2 (**h**), and Alx1 (**i**). Each gene name is near the 5′-end of the gene, and CpG islands are indicated as green boxes beneath each gene. Chromatin states are colored as in Fig. 1b.

Bivalent enrichment is particularly strong among extremely tissue-specific TFs. Seventy-five TFs had tissue specificity scores higher than 6, meaning their expression in their primary tissue is as high as in all other tissue combined (see Methods); of these, 62 were bivalent. Tissue-specific gene expression (Fig. 4a) and chromatin state assignments around eight examples TFs (Fig. 4b–i) reveal distinct modes of gene repression. We highlight one pair of paralogous TFs (Fig. 4d, e). *Gata4* is a bivalent gene that regulates cardiac development^50^; it is primarily expressed in the heart with low expression in the gastrointestinal tract. Accordingly, its TSS exhibits broad Tss states in the heart, narrow Tss regions surrounded by TssBiv and ReprPC regions in the stomach and intestine, and only TssBiv and ReprPC regions in other tissues (Fig. 4e). *Gata4*’s paralog *Gata1* is a key regulator of erythrocyte development^51^ and is predominantly expressed in the fetal liver, but interestingly it is a non-bivalent gene: its TSS exhibits a broad Tss domain in the liver and a narrow Tss domain during early timepoints of the heart, but is labeled Quies in other tissues (Fig. 4d). Thus, there are two distinct TSS modes for gene repression: bivalent and quiescent TSSs.

Other bivalent TFs also exhibit tissue-specific chromatin state patterns. The homeobox-containing TF *Dlx1* is required for the migration of GABAergic neuron progenitor cells from the subcortical telencephalon to the neocortex^52^; it is expressed in the forebrain and facial prominence where its TSS adopts the Tss state, but is repressed and adopts the TssBiv-ReprPC repressive states in other tissues (Fig. 1e). *Arx* is another homeobox-containing TF with a similar forebrain specificity (Fig. 4b); it is important for the maturation and migration of GABAergic interneurons, and its loss-of-function causes lissencephaly (smooth brain) in humans^53^. *En2* encodes a homeobox TF expressed at high levels in Purkinje cells functioning as a transcriptional repressor in neurodevelopment^54^; it is specifically expressed in the midbrain and hindbrain with corresponding tissue-specific chromatin patterns (Fig. 4c). Wilms’ tumor-1 (*WT1*), which encodes a TF and RNA-binding protein, is essential for kidney development^55^. It is predominantly expressed in the kidney and at lower levels in the heart, stomach, and intestine; its TSS is in the Tss state in the kidney, exhibits a broad TssBiv domain in the heart, and is TssBiv-ReprPC in other tissues (Fig. 4f). The forkhead TF *Foxq1* is required for the maturation of mucin-producing foveolar cells in the developing gastrointestinal tract^56^; it is expressed and exhibits the Tss state specifically in these tissues, and is bivalent in other tissues (Fig. 4g). *Evx2* is required for the morphogenesis of limbs^57^, consistent with its expression and chromatin pattern (Fig. 4h). Finally, the aristaless-like homeobox 1 TF *Alx1* has an important role in the development of craniofacial mesenchyme, the first branchial arch, and the limb bud; loss-of-function causes severe disruption of early craniofacial development in humans^58^. Consistent with its functions, *Alx1* is predominantly expressed in the embryonic facial prominence and shows the corresponding chromatin profile (Fig. 4i).

### Genomic regions assigned to TssBiv are highly conserved evolutionarily

**Fig. 5:**
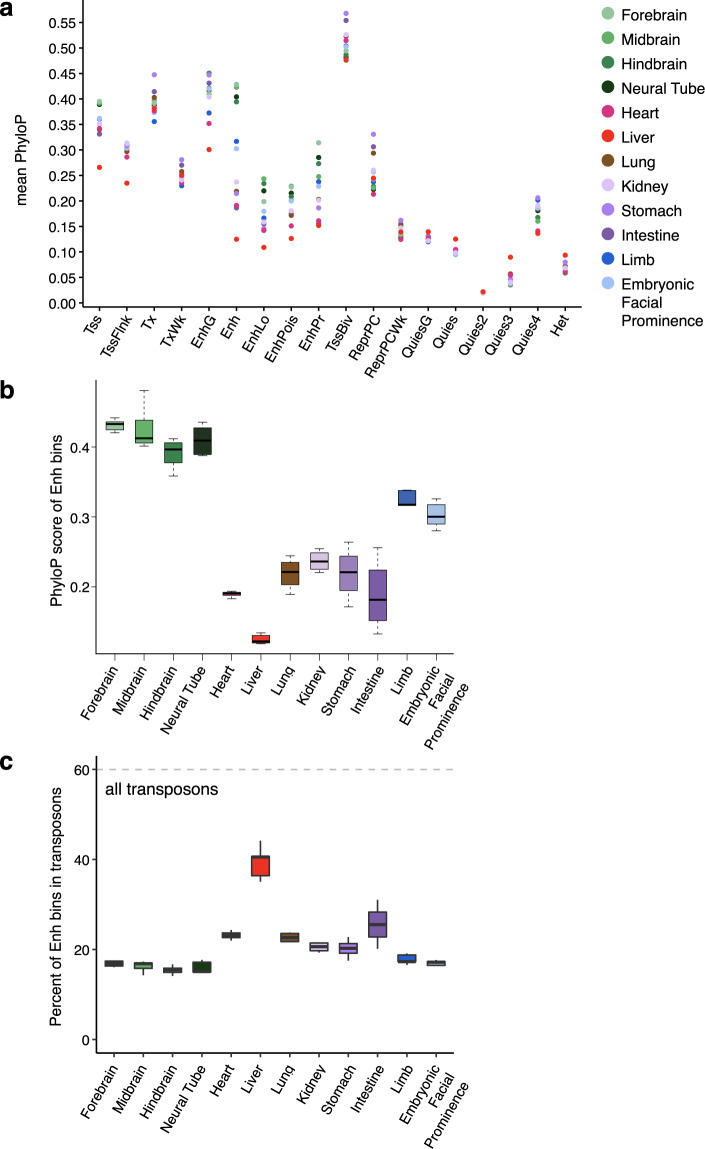
Evolutionary conservation of genomic regions by chromatin state. **a** The PhyloP conservation score (phyloP60way for mm10) for genomic regions assigned to each chromatin state. Colors correspond to tissues. **b** PhyloP score for genomic bins assigned to Enh in all 12 tissues. **c** Percentage of bins assigned to Enh that overlap with transposons, for all 12 tissues.

TssBiv genomic bins are much more evolutionarily conserved than bins assigned to the other 17 chromatin states (Fig. 5a). In each biosample, we calculated the mean PhyloP^59^ score in each genomic bin and then averaged these PhyloP scores across the bins assigned to each chromatin state (see Methods). TssBiv’s PhyloP score (0.51 averaged over the 66 biosamples) was substantially higher (Wilcoxon signed-rank test *P* values < 2.2 × 10^−16^) than the transcription-related states Tx (0.41) and EnhG (0.42), the active-TSS state Tss (0.36), and the high-signal enhancer state Enh (0.30), which were in turn substantially higher than the remaining 13 states. Quies2 (0.02) was the lowest (Fig. 5a).

Enhancer-related regions (Enh, EnhLo, EnhPois, and EnhPr) were most conserved in the four brain tissues (forebrain, midbrain, hindbrain, and neural tube) and least conserved in the liver (Fig. 5a), even considering some variations between timepoints (Fig. 5b, Supplementary Fig. 10). For example, the average PhyloP score of Enh genomic bins was 0.42 for midbrain, whereas it was 0.13 for liver (Wilcoxon rank-sum test *P* value = 5.8 × 10^−4^ for comparing the seven midbrain timepoints with the seven liver timepoints). We examined the transposon content in these Enh genomic bins and found that 40.6% of the Enh genomic bins in the liver overlapped annotated transposons, whereas only 14.1–17.5% of those in the four brain tissues did (Fig. 5c), explaining their substantially different evolutionary conservation. The prevalence of low-conservation, transposon-overlapping liver enhancers is due to the global hypomethylation of the liver genome during hematopoiesis at E11.5 to E16.5^14^.

We directly examined the evolutionary conservation of the TSSs of TFs, stratified by whether they resided in a TssBiv genomic bin or not (the two bottom-right panels in Supplementary Fig. 10). The average PhyloP score of the TF TSSs in TssBiv genomic bins was 0.82, substantially higher than that of the TF TSSs not in TssBiv genomic bins (0.53, Wilcoxon rank-sum test *P* value < 2.2 × 10^−16^ for comparing the two groups in 66 biosamples). Combined with our aforementioned findings that TFs are highly enriched in bivalent regions, these results indicate that TFs with bivalent TSSs have a key role in evolutionarily conserved pathways driving development.

### Genomic regions assigned to TssBiv are enriched in PRC2-bound silencers and their target TSSs

We used a set of 1800 silencers bound by Polycomb Group 2 proteins (PRC2), identified using ChIA-PET assays targeting PRC2 component proteins in mouse embryonic stem cells^33^, to further annotate our chromatin states. The PRC2-bound silencers overlapped extensively with our 14,558 bivalent regions (defined as TssBiv genomic bins surrounded by repressive bins; see Methods): 1069 out of the 1800 silencers overlapped bivalent regions by at least 50% of the lengths of the silencers, whereas, on average, only 21 silencers overlapped with random regions with matching sizes as the bivalent regions (*Z* score = 140; *P* value < 2.2 × 10^−16^). In individual biosamples, centerpoints of most silencers fall within TssBiv or ReprPC bins (24 ± 4% and 28 ± 6% of silencer centers, corresponding to 85.7- and 36.4-fold enrichment over the genomic footprints of these states). This is consistent with the enrichment of these two states in H3K27me3, the histone mark that PRC2 recognizes specifically.

**Fig. 6:**
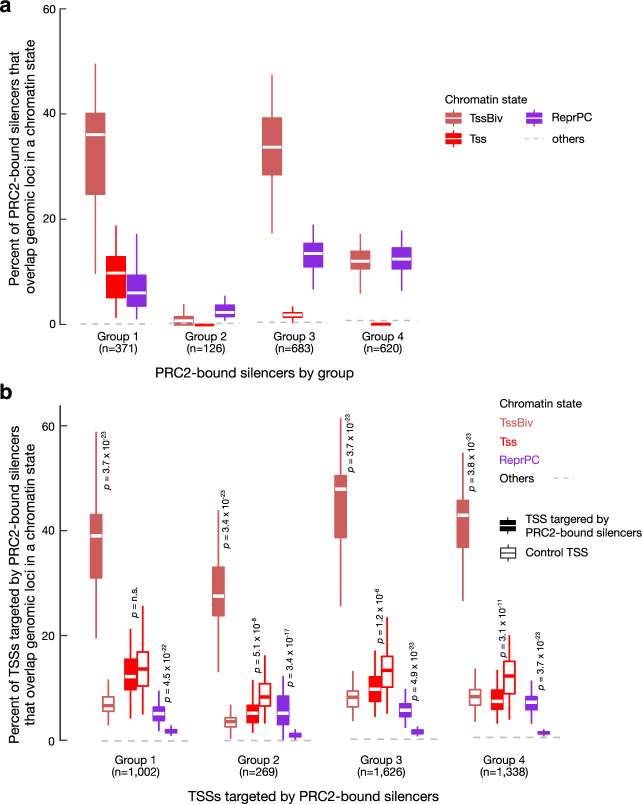
PRC2-bound silencers and their target TSSs are enriched in the TssBiv and ReprPC states. **a** Percentage of PRC2-bound silencers whose centers overlap a genomic bin assigned to the TssBiv, Tss, ReprPC, or other chromatin states. Silencers were divided into four groups by Ngan et al.^33^ according to H3K27ac signals in mouse fetal tissue biosamples. To normalize for the differential genomic coverage of the chromatin states, matching numbers of genomic bins were randomly drawn from the other states to match the number of genomic bins in TssBiv in each biosample. States are colored as in Fig. 1b and the average of the other 15 states is shown as a gray dashed line. **b** Same as **a** but for the TSSs targeted by the PRC2-bound silencers defined by Ngan et al.^33^. We randomly selected TSSs with the same distance distribution from PRC2-bound silencers as a control (open boxplots).

The PRC2-bound silencers were clustered into four groups according to their H3K27ac signal profiles across the fetal mouse tissues^33^, and these groups are enriched in different chromatin states. Group 1 silencers (*N* = 371) had the highest H3K27ac signals in the fetal mouse tissues^33^, and their centerpoints fell within Tss and Enh states in some biosamples, especially in the brain but not in the liver (Supplementary Fig. 11). Group 2 silencers (*N* = 126) were depleted in H3K27ac in all fetal mouse tissues^33^, and their centers fell in quiescent states in all tissues. Group 3 and 4 silencers (*N* = 683 and 620) had intermediate levels of H3K27ac (higher in Group 3 than in Group 4)^33^, and their centers mostly fell in TssBiv and ReprPC states. We included in these alluvial plots chromatin state assignments in mouse embryonic stem cells (ES-Bruce4) produced with our 18-state, ten-mark model; overall, these show similar chromatin state assignments as in the fetal tissues (see Methods and Supplementary Fig. 11; note that H3K4me2, ATAC, and WGBS data are not available for embryonic stem cells). To normalize for the genomic footprint of each genomic state, we compared genomic bins assigned to TssBiv (the least abundant state; Fig. 1c) with an equal number of genomic bins randomly drawn from the other states in individual biosamples for their overlap with PRC2-bound silencers. TssBiv showed the highest enrichment for Group 1 and Group 3 silencers and moderate enrichment for Group 4 silencers; ReprPC showed moderate enrichment for all groups of silencers; Tss showed moderate enrichment for only Group 1 silencers; and no other states showed enrichment (Fig. 6a).

ChIA-PET data further provided target TSSs for each PRC2-bound silencer^33^, and these also predominantly fell in TssBiv, Tss, and ReprPC states. The most active Group 1 silencer had the highest percentage of active target TSSs of the four subtypes (Supplementary Fig. 12), a pattern again most prevalent in the brain and least in the liver (57.9% and 13.4% for forebrain and liver; Supplementary Fig. 12). After normalizing for the genomic footprints of the chromatin states, TssBiv showed a strong enrichment for the target TSSs of all groups of silencers, whereas Tss and ReprPC showed weak enrichment (Fig. 6b). We further controlled for the distances between the PRC2-bound silencers and their target TSSs by randomly drawing non-target TSSs with the same distance distribution from the PRC2-bound silencers; as expected, the Tss state is not enriched for this control (it is even significantly depleted for Groups 2, 3, and 4), but TssBiv and ReprPC remain significantly enriched (Fig. 6b). Among the 75 tissue-specific TFs (Fig. 4a), 44 of the 62 bivalent TFs but none of the 13 non-bivalent TFs were targeted by the PRC2-bound silencers (Fisher’s exact *P* value = 1.6 × 10^−6^). Five of the seven example bivalent TFs (Fig. 4b–i) were targeted by the silencers (*En2*, *Gata4*, *Wt1*, *Foxq1*, and *Evx2*).

## Discussion

We defined 18 chromatin states by integrating data on eight histone marks, chromatin accessibility, and DNA methylation in 66 biosamples across fetal mouse development (Fig. 1). We recapitulated the human states previously defined by the Roadmap Epigenomics Consortium^10^ and refined enhancer, bivalent, and quiescent states. Regions belonging to these states varied more among tissues at the same developmental timepoint than across timepoints in the same tissue (Fig. 2a), and the variations in the Enh and TssBiv regions were specific enough to distinguish the tissue-of-origin for the 66 biosamples (Fig. 2d). Our chromatin state annotation should provide a useful resource for studying mammalian development.

Because enhancers and promoters have been examined extensively in previous ChromHMM studies^6,10,34^, we focused instead on the TssBiv state. TssBiv has the smallest genomic footprint among the 18 states (~0.3% of the genome in any particular biosample), yet is discovered consistently by the five-mark, eight-mark, and ten-mark models and is the most conserved evolutionarily of any state (Fig. 5). We define 14,558 bivalent regions upon integration of data in 66 biosamples, of which roughly half overlap GENCODE-defined TSSs; these bivalent TSSs are repressed in a tissue-specific manner (Fig. 3) and are frequently tissue-specific TF genes (Figs. 3, 4). Interestingly, bivalent TF TSSs are significantly more conserved than their non-bivalent counterparts (Supplementary Fig. 10, bottom right). Comparison with recent ChIA-PET data^33^ revealed that bivalent regions are also enriched in PRC2-bound silencers and their target TSSs, and conversely, the TSSs of the target genes of PRC2-bound silencers are highly enriched in the TssBiv state in individual biosamples (Fig. 6).

Both PRC2-bound silencers and their target TSSs possess the same epigenetic signature and hence are assigned the same TssBiv state. This is perhaps not surprising because they are bound by the PRC, which drives the formation of a three-dimensional network including low-expression genes with bivalent promoters^60^. Despite being transcriptionally repressed, bivalent regions lie within open chromatin regions that associate with the active compartment^61,62^. When embryonic stem cells differentiate, bivalent gene activation displaces Polycomb proteins and disrupts the three-dimensional interactions among Polycomb-bound regions^60,63^. Thus, PRC2-bound silencers and their target TSSs are likely to be in spatial proximity and hence share the same epigenetic signature. Along this line of reasoning, Enh and Tss states (Fig. 1c) also share some epigenetic features (open chromatin, high levels of active histone marks, and low DNA methylation).

Our systematic analysis of bivalent regions in mouse fetal tissues complements earlier studies on bivalent regions in other cell types and biological systems. Bivalent regions were first discovered in embryonic stem cells^23^, where their functions have been extensively studied. They have been shown to repress their associated genes, which were found to be enriched in developmental TFs, and yet allow them to be poised for quick responses to stimuli^23,44^. When embryonic stem cells differentiate, these bivalent genes become monovalent, retaining either the active marks or the repressive mark, and accordingly being expressed or repressed^19^. Subsequent studies reported bivalent domains in differentiating CD4+ T cells^30^, the multipotent cranial neural crest cells^29^, adult cells in intestinal villi with regenerative potential^26^, and terminally differentiated medium spiny neurons in the striatum^27^. In each study, disruption of Polycomb group proteins led to the activation of bivalent genes but not genes marked by H3K27me3 only^26,27^, suggesting that bivalency is a mechanism for persistent gene repression from embryonic stem cells to terminally differentiated cells.

Our analysis of bivalent genes indicates that they have low expression levels in the mouse fetal tissues where they are bivalent and are enriched for developmental TFs under tissue- and timepoint-specific repression. A repressed gene can be in a quiescent chromatin state, which corresponds to low levels of all histone marks and high DNA methylation, such as *GATA1* (Fig. 4d). Alternatively, it can be in an H3K9me3-enriched Het state accompanied by low levels of active histone marks and high levels of DNA methylation. However, a majority of the bivalent TSSs in fetal tissues overlap CpG islands (mean = 62.5% across the 66 biosamples, vs. 29.8% for non-bivalent TSSs). DNA-hypomethylated CpG islands recruit both Polycomb group and Trithorax group proteins to lay down H3K27me3 and H3K4me3 marks respectively, and the expression level of the gene reflects the competition between Polycomb-mediated repression and Trithorax-mediated activation^64–66^. As a result, the interplay between the TssBiv, Tss, and ReprPC chromatin states (Supplementary Fig. 3b) reflects the main mechanism—distinct from quiescent or Het chromatin states—for silencing genes with CpG-rich TSSs in a tissue-specific manner throughout fetal development and possibly in adulthood.

One limitation of our chromatin state assignments is the heterogeneity of cells in a tissue sample. We cannot distinguish a scenario where a region has low signal across all cell types in a tissue from one where the region has high signals in a small subset of cells and no signal in the remaining cells. This limitation may be of particular relevance for the quiescent states that have low levels of H3K9me3 and H3K27me3 signals, which could correspond to heterochromatic regions in a subpopulation of cells.

In conclusion, we present genome-wide annotations of 18 chromatin states using ten chromatin marks all assayed in twelve mouse fetal tissues across 4–7 developmental timepoints at daily intervals from E11.5 to birth. These comprehensive annotations enabled us to investigate the changes of chromatin profiles across tissue and timepoints. We analyzed bivalent regions in detail and found these evolutionarily conserved regions to be highly enriched in master transcriptional factors important for regulating tissue-specific developmental processes. More broadly, our results suggest that bivalent regions represent a mechanism for silencing CpG-rich genes in a tissue- and timepoint-specific manner.

## Methods

### Experimental data processing for mouse epigenome construction and chromatin state definition

We downloaded data sets processed for the mouse genome (mm10) from the ENCODE Portal^12,67^ (http://encodeproject.org) that corresponded to eight histone marks (H3K4me1, H3K4me2, H3K4me3, H3K9ac, H3K27ac, H3K36me3, H3K9me3, H3K27me3), ATAC-seq, and WGBS for each of 66 epigenomes (Supplementary Data 1). All biosamples were from the C57BL/6 mouse strain. For each histone mark, two biological replicates of the ChIP experiment were performed, and for each epigenome, two replicates of the control (input) experiment were performed. We ran ChromHMM^6^ on the 66 epigenomes at the default 200-bp resolution, assigning each 200-bp genomic bin (13,627,678 of them in total for the entire mouse genome) to a chromatin state in each biosample. We used the histone ChIP-seq BAM files and the relevant control files for each data set. For ATAC-seq data, each BAM file was converted to a signal track as follows. Reads were extended to their fragment size and counts-per-million were calculated for all non-overlapping 200-bp genomic bins. Quantile normalization was then applied across the entire data set and the normalized signal was binarized, using a threshold of 0.5. For WGBS data, BED files were downloaded from the ENCODE portal (Supplementary Data 1), These files contain, among other values, the percent methylation at each CpG dinucleotide in the genome (ranging from 1–100). For each set of two replicates, these values were averaged in 200-bp genomic bins to obtain the mean percent methylation of CpGs in each window. The 200-bp bins were subsequently binarized based on a 50% methylation threshold. Bins that did not contain any CpGs were marked as missing data, as specified by the ChromHMM binarized data format.

We defined 18 chromatin states using ChromHMM^6^ using the processed data described above on the 10 marks. We used the genomic bins with posterior probability >0.5 for the downstream analysis; these bins composed 97.1% (±0.53%) of the genome across the tissue samples.

For comparison, we also made two sets of 15-chromatin-state assignments using a subset of the data: a five-mark, 15-state model using five histone marks (H3K4me1, H3K4me3, H3K36me3, H3K9me3, H3K27me3), and an eight-mark, 15-state model using all eight histone marks. The eight-mark, 15-state model is similar to the ChromHMM model recently published by Gorkin et al.^13^, with the following correspondence of states between our model and those of Gorkin et al.:

Tss: Active Promoter (Pr-A, State 1)

TssFlnk1: Strong Enhancer, TSS proximal (En-Sp, state 6)

TssFlnk2: Weak/inactive Promoter (Pr-W, state 2)

Tx: Strong Transcription (Tr-S, state 10)

EnhG: Initiation Transcription (Tr-I, state 12)

Enh: Strong Enhancer, TSS-distal (En-Sd, state 5)

EnhLo1: N/A

EnhLo2: Weak Enhancer, TSS-distal (En-W, state 7)

EnhPois1: Poised Enhancer, TSS proximal (En-Pp, state 9)

EnhPois2: Poised Enhancer, TSS-distal (En-Pd, state 8)

TssBiv: Bivalent Promoter (Pr-B, state 3)

ReprPC: Polycomb-associated heterochromatin (Hc-P, state 13)

QuiesG: Permissive Transcription (Tr-P, state 11)

Quies: No signal (Ns, State 15)

Het: H3K9me3-associated heterochromatin (Hc-H, state 14)

N/A: Flanking Promoter (Pr-F, state 4)

### Enrichment of chromatin states in other annotations (Fig. 1c)

We assessed the chromatin state assignments in each of the 66 epigenomes for their enrichments in three types of annotations (Fig. 1c, the right panel titled Enrichment): (1) for CpG islands, we downloaded cpgIslandExtUnmasked.txt from the UCSC Genome Browser; (2) we used GENCODE version M4 for gene-related annotations (TSS, transcription end sites or TES, gene, exon, and intron); and (3) we used epigenetic annotations (EP300 and CTCF ChIP-seq peaks and DHS).

For every chromatin state, we computed its enrichment for each annotation, defined as the observed joint probability (*P*) of a chromatin state and an annotation occurring together over the expected joint probability (i.e., assuming the state and the annotation occur independently):

$$Enrichment = P( {chromatin\;state_i,\;annotation_j} )/[ {P( {chromatin\;state_i} ) \times P( {annotation_j} )} ]$$

For visualization (the right panel of Fig. 1c titled Enrichment), the enrichments were scaled between 0 and 1:

$$Enrichment_{scaled} = \left( {Enrichment - Enrichment_{\min }} \right)/\left( {Enrichment_{\max } - Enrichment_{\min }} \right)$$

We further integrated the RNA-seq data (Supplementary Data 1) processed with the ENCODE uniform processing pipeline to compute the enrichment of the chromatin states is expressed or repressed genes for each of the 66 epigenomes^12^. For plotting the enrichment panels in Fig. 1c, we clustered genes into either expressed or repressed groups in each biosample based on an expression level cutoff determined using a two-component Gaussian mixture model. The expression levels (in TPM) for the two replicates of each biosample were averaged.

We calculated the enrichment of the chromatin states in EP300 and CTCF ChIP-seq peaks and DHS (the right-most panel in Fig. 1c) for those epigenomes that had the EP300 and CTCF ChIP-seq or DNase-seq data available in the corresponding tissues and timepoints (Supplementary Data 1). For the EP300 ChIP-seq data, the BAM files from two biological replicates were pooled, and peaks were called using MACS2^68^ with the q-value cutoff of 0.01. For the CTCF ChIP-seq data, the optimal IDR thresholded peaks^69^ defined by the ENCODE uniform ChIP-seq pipeline were used^12^. For the DNase-seq data, the hotspots defined by the ENCODE uniform DNase-seq processing pipeline were used^12^.

### Partial epigenome simulation and construction (Fig. 1d)

To assess the reliability of chromatin state assignments on epigenomes that lacked the data for one of the ten chromatin marks, for each biosample we simulated ten partial epigenomes, starting with the ten-mark epigenome and omitting the data for each mark individually. We applied the ten-mark 18-state ChromHMM model to the available data on the remaining nine marks and compared the resulting chromatin states assignments with the chromatin state assignments of the ten-mark epigenome by computing the Jaccard similarity between all genomic bins (Fig. 1d). The chromatin states with Jaccard similarity <0.5 were labeled as misassigned in the missing-one-mark epigenomes.

For the comparison with PRC2-bound silencers in embryonic stem cells, we also performed chromatin state assignment on embryonic stem cells, with data on seven histone marks (Supplementary Data 1), missing H3K4me2, ATAC, and DNA methylation data. We simulated the effect of missing three marks using midbrain and forebrain samples and concluded that they did not have a major impact on the assignment of the TssBiv state. The chromatin state assignments of the seven-mark epigenomes were used to define bivalent genes in mouse embryonic stem cells and compared with the bivalent genes in mouse fetal tissues defined using the chromatin state assignments of the ten-mark epigenomes (see below).

### Chromatin state variations across tissues and timepoints (Fig. 2a)

We computed Jaccard similarity between a pair of epigenomes by comparing the chromatin states at the corresponding genomic bins between the two epigenomes.

### UMAP analysis of the epigenomes (Fig. 2d)

We performed two-dimensional visualization of the 66 epigenomes using UMAP^40^ analysis on two sets of 200-bp genomic bins: those assigned to the Enh state or the TssBiv state in one or more biosamples. For the Enh genomic bins, UMAP was provided with the H3K27ac signal levels across the 66 biosamples and the following parameters were used: n_neighbors = 7, min_dist = 0.5, seed = 11. For the TssBiv genomic bins, UMAP was provided with the signal levels of all ten marks across the 66 biosamples and the following parameters were used: n_neighbors = 10, min_dist = 0.04, seed = 12.

### Identification of bivalent TSSs and bivalent genes (Figs. 3, 4, Supplementary Figs. 4–8)

We developed a method to identify bivalent TSSs and bivalent genes by their chromatin states in each epigenome, described as follows. We first converted each epigenome to a character string using an 18-letter alphabet (one symbol for each state). Regular expressions were then used to extract punctate (median length 1800 bp) bivalent domains (stretches of contiguous genomic bins) in each epigenome, defined as bivalent chromatin states flanked by quiescent or heterochromatin states (ReprPC, ReprPCWk, Quies, Quies2, Quies3, Quies4, or QuiesG state). We used the union (14,558 regions across all tissue timepoints, median 3514 per tissue timepoint, neighboring regions were not merged) of the detected genomic regions matching our regular expression for downstream analyses. The 14,558 regions detected in the 66 biosamples collectively overlapped 14,729 GENCODE-annotated TSSs; we denote these bivalent TSSs. We further define a bivalent gene as having at least one bivalent TSS, yielding 6797 genes that are bivalent in any of the 12 tissues.

We detected on average ~3400 bivalent genes per tissue, defined as genes that are bivalent in any of the timepoints in the tissue. We performed Gene Ontology (GO) analysis on bivalent genes using the PANTHER tool^70^. The genes used in the GO analysis, of which the results are listed in Supplementary Data 4 were obtained as follows: TSSs extracted from the M4 GENCODE annotations were intersected with the bivalent regions detected in each tissue. For each tissue, genes for which one or more TSSs intersected were retained. Then, the 1077 genes that were found to have TSSs overlapping bivalent regions in all tissues were used as input for the GO analysis (Supplementary Data 4a, b). Another set of 1291 genes was obtained using the same process, except that these genes had TSSs in bivalent regions only in liver samples and not in any other 11 tissues (Supplementary Data 4c, d). Gene IDs were translated into gene names prior to submission to PANTHER. For six gene IDs, no matching gene name was found, leaving 1074 and 1288 genes in the “all tissues” and the “liver-only” gene sets for submission. PANTHER was run on the GO “Biological Process” ontology, using Fisher’s exact test and FDR for *P* value calculations.

We computed the expected number of genes in a tissue that would be bivalent at all seven timepoints if the timepoints were to be independent of one another (Fig. 3a, Supplementary Fig. 6). Using the liver as an example, we computed the product of the frequencies of bivalent genes in each timepoint in the liver (0.64, 0.83, 0.78, 0.68, 0.80, 0.73, and 0.52 for E11.5, E12.5,… P0, respectively), then multiplied this product by the total number of genes bivalent in the liver at one or more timepoints (*N* = 5176). For the calculation of the frequency at each timepoint, we also used the total number of genes bivalent in the liver at one or more timepoints (*N* = 5176) as the denominator.

### Gene annotations and identification of TFs (Fig. 3d, Fig. 4, Supplementary Data 2–4)

GENCODE M4 gene annotations were used to identify genes and TSSs. To avoid double-counting TSSs, coinciding TSSs were merged. To identify TFs, we used the list of TFs and their homologs in mouse and human^47^. Ensembl IDs were obtained by mapping gene names to the GENCODE M4 annotations^71^. In all, 552 TFs matched IDs in the GENCODE M4 mouse annotations.

### Evolutionary analysis (Fig. 5a–b, Supplementary Fig. 10)

We averaged the mouse 60-way phyloP^59^ score across the genomic positions in each 200-bp genomic bin. We then average this per-bin score for all the genomic bins assigned to a particular chromatin state in each biosample to obtain the average PhyloP score per state per biosample (Supplementary Fig. 10, first 18 panels). For each tissue (Fig. 5a), the PhyloP scores from the biosamples at different timepoints were further averaged. For the TF TSSs (Supplementary Fig. 10, the two bottom-right panels), we used the PhyloP score for genomic bins where each TF TSS resided in, stratified by whether that bin was assigned to the TssBiv state or not.

### Overlap of Enh regions with annotated transposons (Fig. 5c)

We used transposon annotations in the mouse genome from Repbase^72^ to analyze the Enh state across different tissues (Fig. 5c). We overlapped the genomic bins assigned to the Enh state in each biosample with annotated transposons, requiring at least one-bp overlap. The percentage of all genomic bins that overlapped transposons was used as control (gray dashed line in Fig. 5c).

### Analysis of PRC2-bound silencers (Fig. 6, Supplementary Figs. 11, 12)

We used the 18,000 PRC2-bound silencers classified into four groups based on their H3K27ac signal in mouse fetal tissues^33^. We overlapped the PRC2-bound silencers with our 14,558 bivalent regions, requiring at least half of the length of a silencer length to overlap. We randomly selected genomic regions with the same lengths as the bivalent regions to act as controls. Furthermore, we assigned each silencer to a chromatin state in a particular biosample according to which chromatin state the center of the silencer falls in.

We included embryonic stem cells in this analysis (ES-Bruce4). These cells were derived from C57BL/6, the same strain of mice from which the tissues were harvested. We only had data on seven histone marks on embryonic stem cells (Supplementary Data 1), and simulation of this partial epigenome (see above Methods) showed no major impact on the assignment of the TssBiv state and the resulting bivalent genes. Simulating using midbrain and forebrain samples, we found that most bivalent genes were identified using the partial epigenome. For example, among the 2250 bivalent genes in the midbrain E11.5 sample, 2014 (89.5%) were identified using the partial epigenome.

### Reporting summary

Further information on research design is available in the Nature Research Reporting Summary linked to this article.

## Supplementary information

Supplementary Information

Description of Additional Supplementary Files

Supplementary Data 1

Supplementary Data 2

Supplementary Data 3

Supplementary Data 4

Reporting Summary

## Data Availability

All experimental data used in this paper can be accessed at the encode Portal (http://www.encodeproject.org/), using the accession IDs listed in Supplementary Data 1. The chromatin state assignments for both the eight-mark, 15-state model and the 10-mark, 18-state model are also accessible at the ENCODE Portal (https://www.encodeproject.org/search/?searchTerm=ChromHMM+Zhiping+Weng). State annotations with posterior probabilities for all 200 bp genomic bins can be found at 10.6084/m9.figshare.13271705^73^. Per-bin PhyloP scores and state durations are available at 10.6084/m9.figshare.13271684^74^. Epigenetic signals of Enh states and TssBiv states for UMAP analysis are available at 10.6084/m9.figshare.13271717^75^. We made a track hub (https://publications.wenglab.org/mouse_epigenomes/trackhub/hub_0.txt) for the UCSC genome browser^76^ to visualize all the data and annotations used in this study listed below. The trackhub can be accessed via a UCSC session: https://genome.ucsc.edu/s/zlab/mouse_epigenomes. 1. Ten-mark, 18-state chromatin state assignments (in dense mode) BigWig experimental data complete for 66 biosamples (in hide mode): a. ChIP-seq of eight histone marks b. ATAC-seq c. WGBS d. RNA-seq e. DNase when available f. EP300 ChIP-seq when available g. CTCF ChIP-seq when available 2. ES-Bruce4 chromatin state assignments (in dense mode) BigWig experimental data for ES-Bruce4 (in hide mode) a. ChIP-seq of seven histone marks b. RNA-seq c. EP300 ChIP-seq d. CTCF ChIP-seq 3. Turn on the GENCODE gene annotation (in pack mode) 4. Turn on the CpG island track from UCSC (in dense mode) 5. Bivalent regions (in dense mode) 6. PRC-bound silencers and their target TSSs in two tracks (in dense mode) 7. Turn on the PhyloP conservation track (in full mode) 8. Turn on VISTA enhancer track hub (in hide mode) 9. Mouse cCREs defined by the ENCODE consortium^12^ (in hide mode)

## Code availability

The code used to extract genomic regions based on regular expression can be found at 10.5281/zenodo.4448387^77^.
