# Supplementary material for: Annotation of chromatin states in 66 complete mouse epigenomes during development
Source: Commun Biol. 2021 Feb 22;4:239. doi: 10.1038/s42003-021-01756-4 (PMC7900196; doi:10.1038/s42003-021-01756-4)
Supplement: Supplementary file 1 — Supplementary Information [file 42003_2021_1756_MOESM1_ESM.pdf]

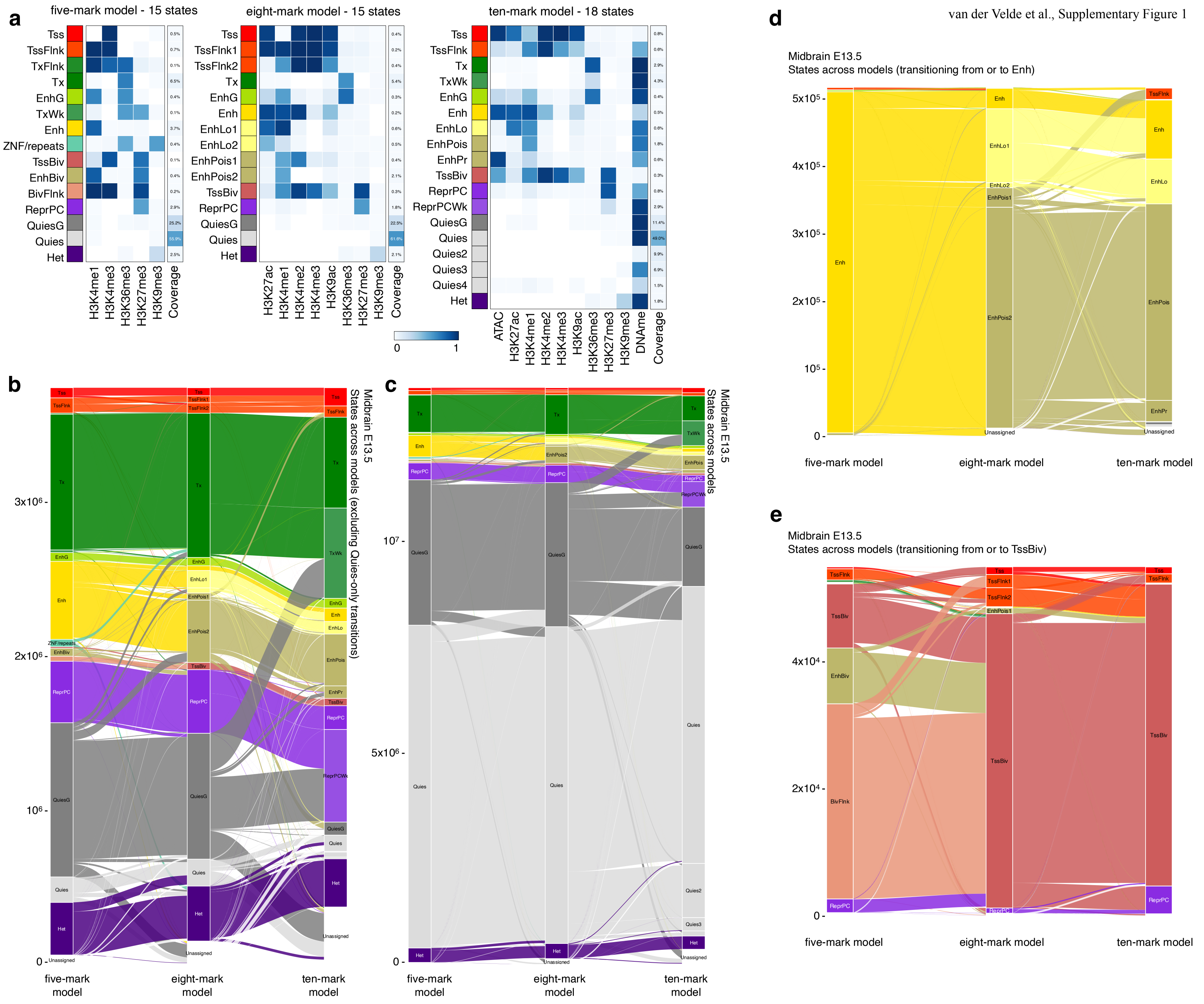

Supplementary Fig. 1: Comparison of the five-mark, eight-mark, and ten-mark models.
a Emission probabilities for the five-mark 15-state model, the eight-mark 15-state model, and the ten-mark 18-state model, with the ten-mark model reproduced from **Fig. 1c** for easy comparison with the other two models. **b-e** Alluvial plots illustrate the correspondence of chromatin states across the three models in forebrain e13.5. **b** With genomic bins assigned to a quiescent state by all three models omitted, 3,743,342 genomic bins are shown. **c** All 13,627,678 200-bp bins in the genome. **d** Genomic bins assigned to Enh by one of the models. **e** Genomic bins assigned to TssBiv by one of the models.

van der Velde et al., Supplementary Figure 2

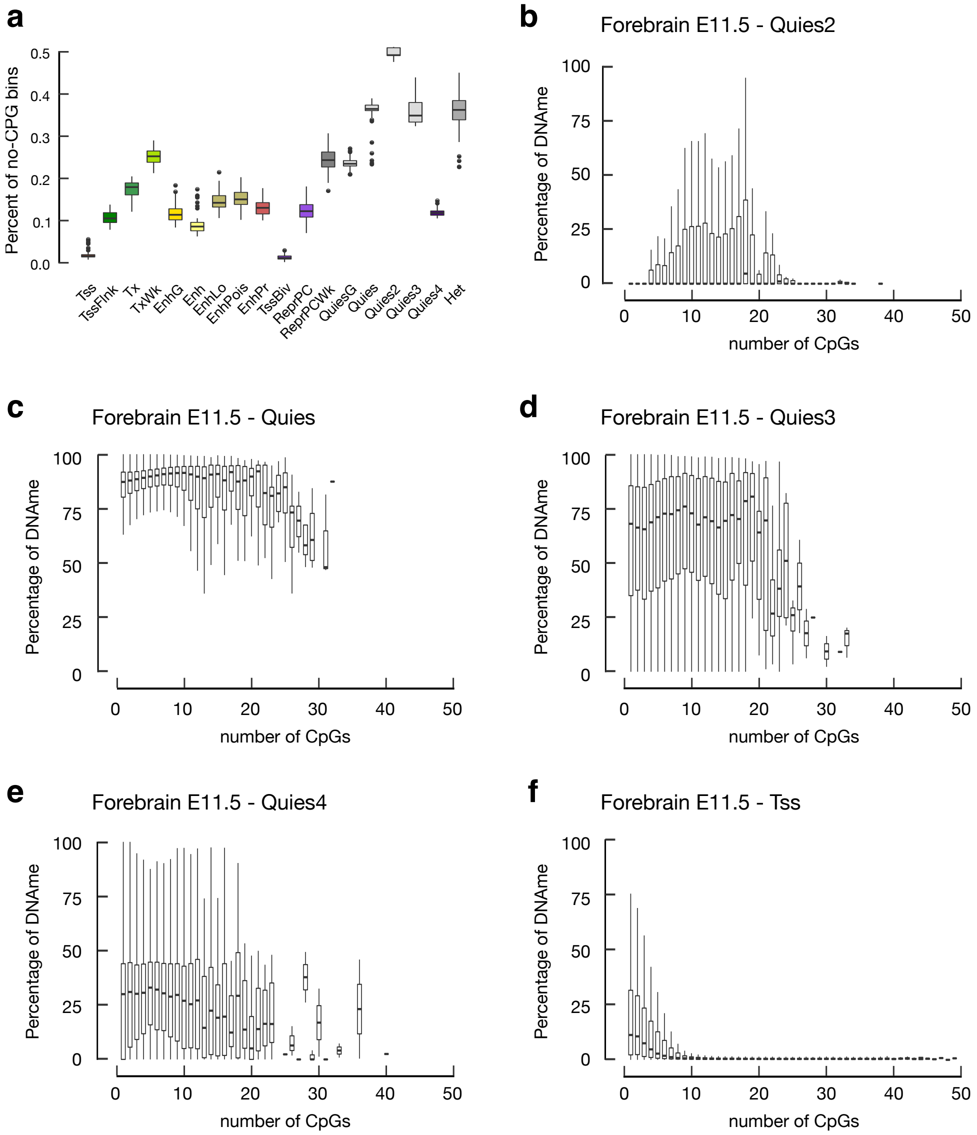

Supplementary Fig. 2: DNA methylation level of genomic bins with different number of CpGs.
a Boxplots show that different states with different percents of no-CpG bins. **b-f** Boxplots show the percent of DNAme of bins with different numbers of CpGs in state Quies2 **(b)**, Quies **(c)**, Quies3 **(d)**, Quies4 **(e)**, and Tss **(f)**. For all box-plots, whiskers show 95% confidence intervals, boxes represent the first and third quartiles, the vertical midline is the median, and outliers are omitted.

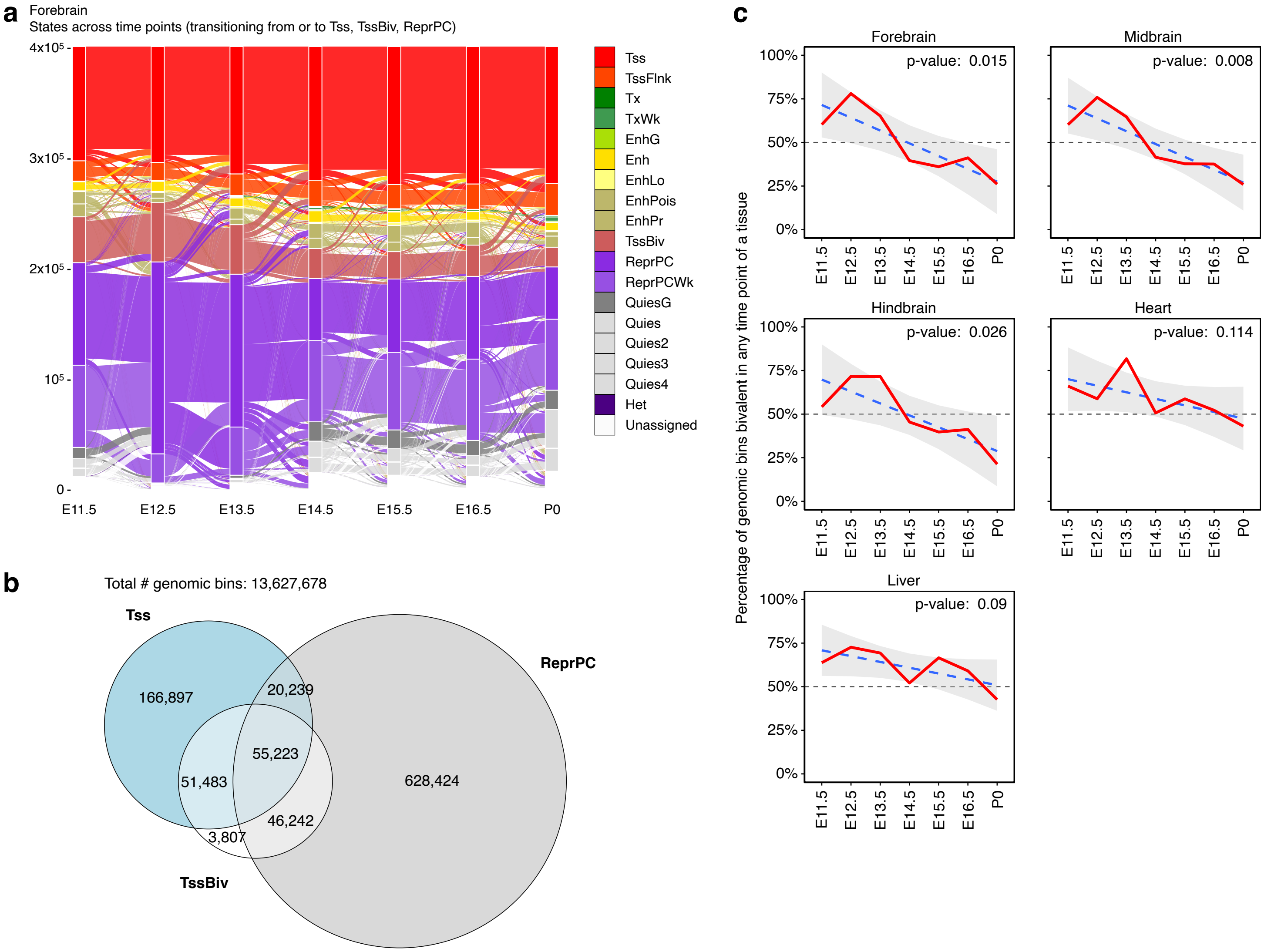

Supplementary Fig. 3: Chromatin state transition from early to late time-points.
a Genomic bins assigned to TssBiv in the forebrain at one or more time-points are included. States are colored as in Fig. 1c. Panels b and c show comparison of genomic coverages of TssBiv, Tss and ReprPC, and the decrease of TssBiv coverage over time, **b** A Venn diagram shows the overlap of genomic regions assigned to the TssBiv, Tss, or ReprPC state in any of the 66 epigenomes. **c** red lines show the percentages of the genome in the TssBiv state over the time course of development for each tissue. Only the five tissues with seven time-points are included. P-values for linear fit (blue dashed line, with the 95% confidence interval in gray shaded area) are provided.

a

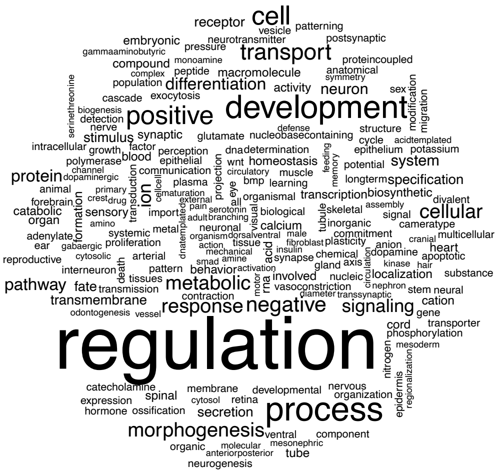

Bivalent TSS in all tissues

b

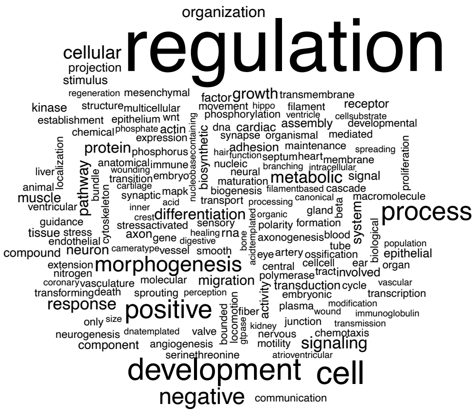

Bivalent TSS only in liver

Supplementary Fig. 4: Word clouds for enriched GO terms in bivalent genes. Gene ontology (GO) enrichment analyses were performed using the PANTHER tool for two groups of genes: genes with bivalent TSSs in **(a)** all 12 tissues; **(b)** in the liver and not in any other tissues. For each analysis, a summary of significantly enriched GO terms is presented as a word cloud. See Supplementary Table 4 for full PANTHER results.

van der Velde et al., Supplementary Figure 5

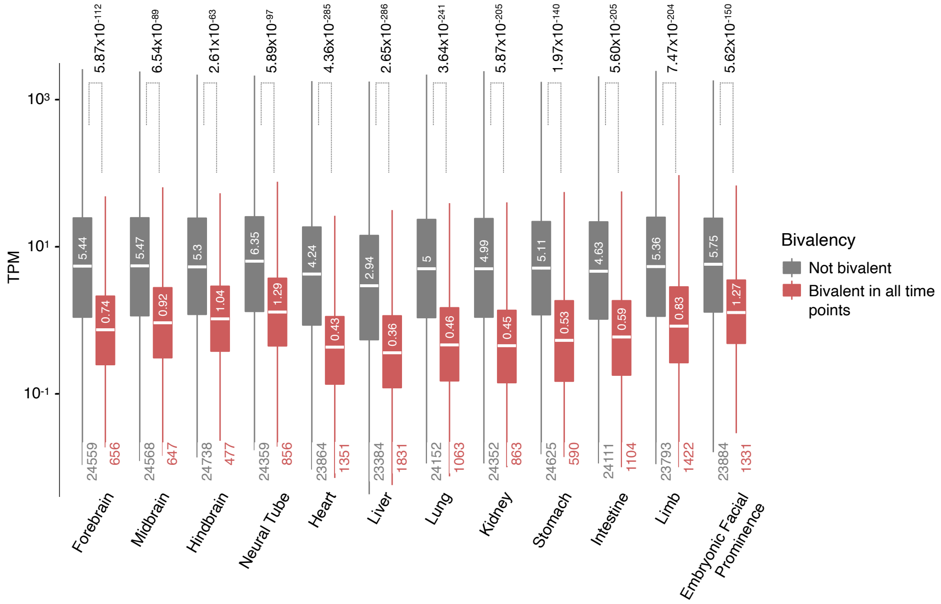

Supplementary Fig. 5: Expression levels of genes with or without bivalent TSSs.
For each tissue, the expression levels of genes (in TPM) are plotted, stratified by whether it has a bivalent TSS at all time-points. For each box plot, the total number of genes in each group is shown at the bottom. Outliers are omitted for clarity. Two-sided Wilcoxon P-values for comparing the two groups of genes in each tissue are provided. For all box-plots, whiskers show 95% confidence intervals, boxes represent the first and third quartiles, the vertical midline is the median, and outliers are omitted.

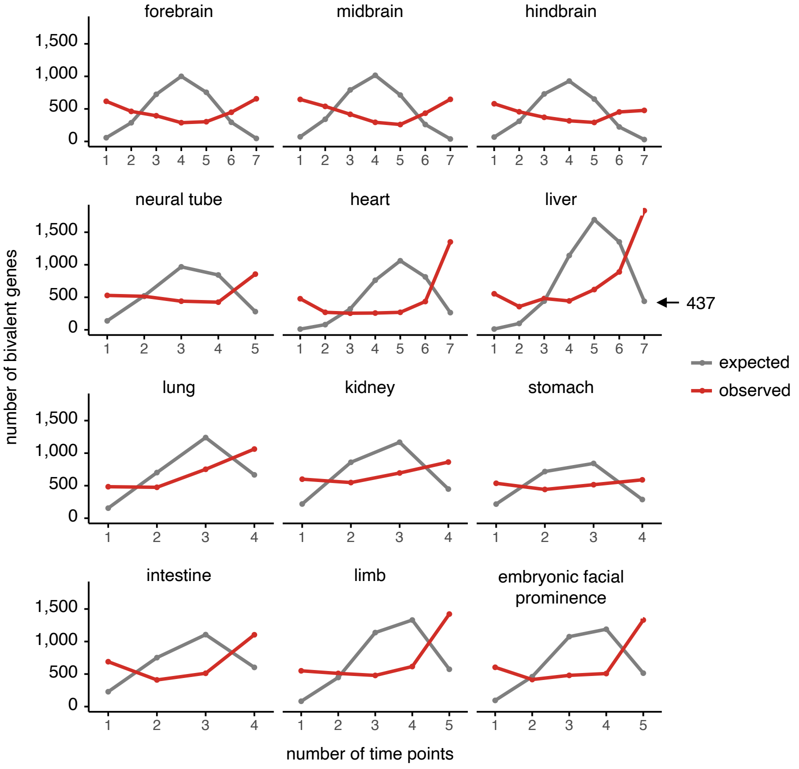

Supplementary Fig. 6: Number of genes that are bivalent in a certain number of time-points. For each tissue, the total number of genes that are deemed bivalent in a certain number of time-points is plotted in red, compared with the expected number (in grey) if genes were randomly assigned to be bivalent at each time-point.

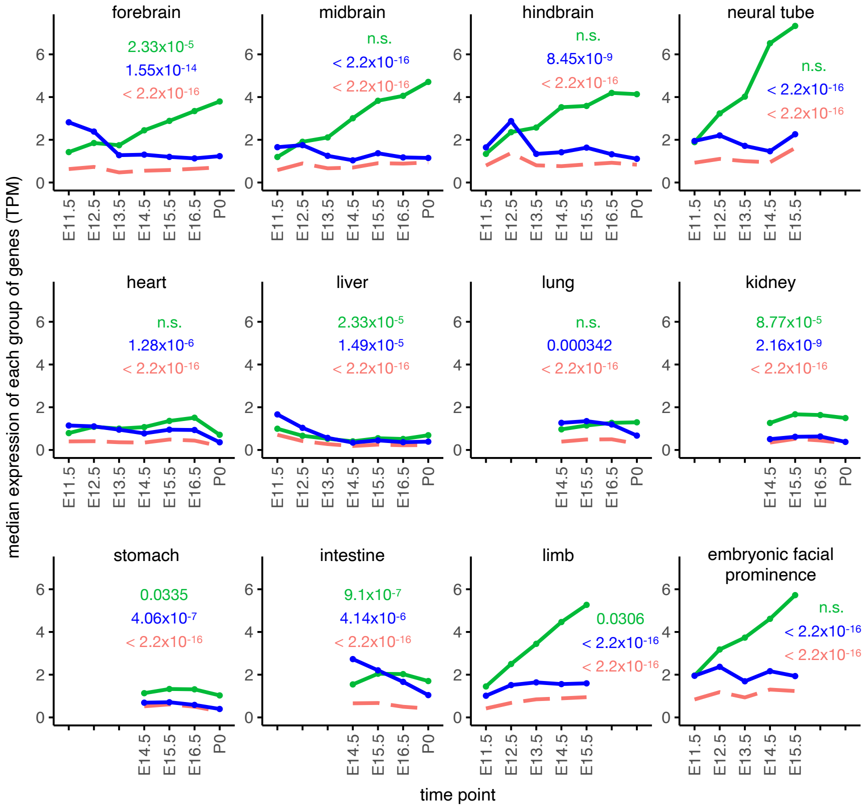

Supplementary Fig. 7: Expression of genes with bivalent TSS at early vs. late time-points.

Median expression levels of genes stratified into three distinct categories are plotted: genes deemed bivalent at the first time-point but not at the last (early-bivalent genes; blue line); genes deemed bivalent at the last time-point but not at the first (late-bivalent genes; green); and genes with bivalent TSS at all time-points (all-bivalent genes; red dashed line). Two-sided Wilcoxon rank-sum test P-values for the comparisons between early- and late-bivalent genes for their expression levels at the first time-point (green P-values); between the early- and late-bivalent genes at the last time-point (blue P-values); and between all-bivalent genes vs. early- and late-bivalent genes (red P-values). n.s. stands for not significant.

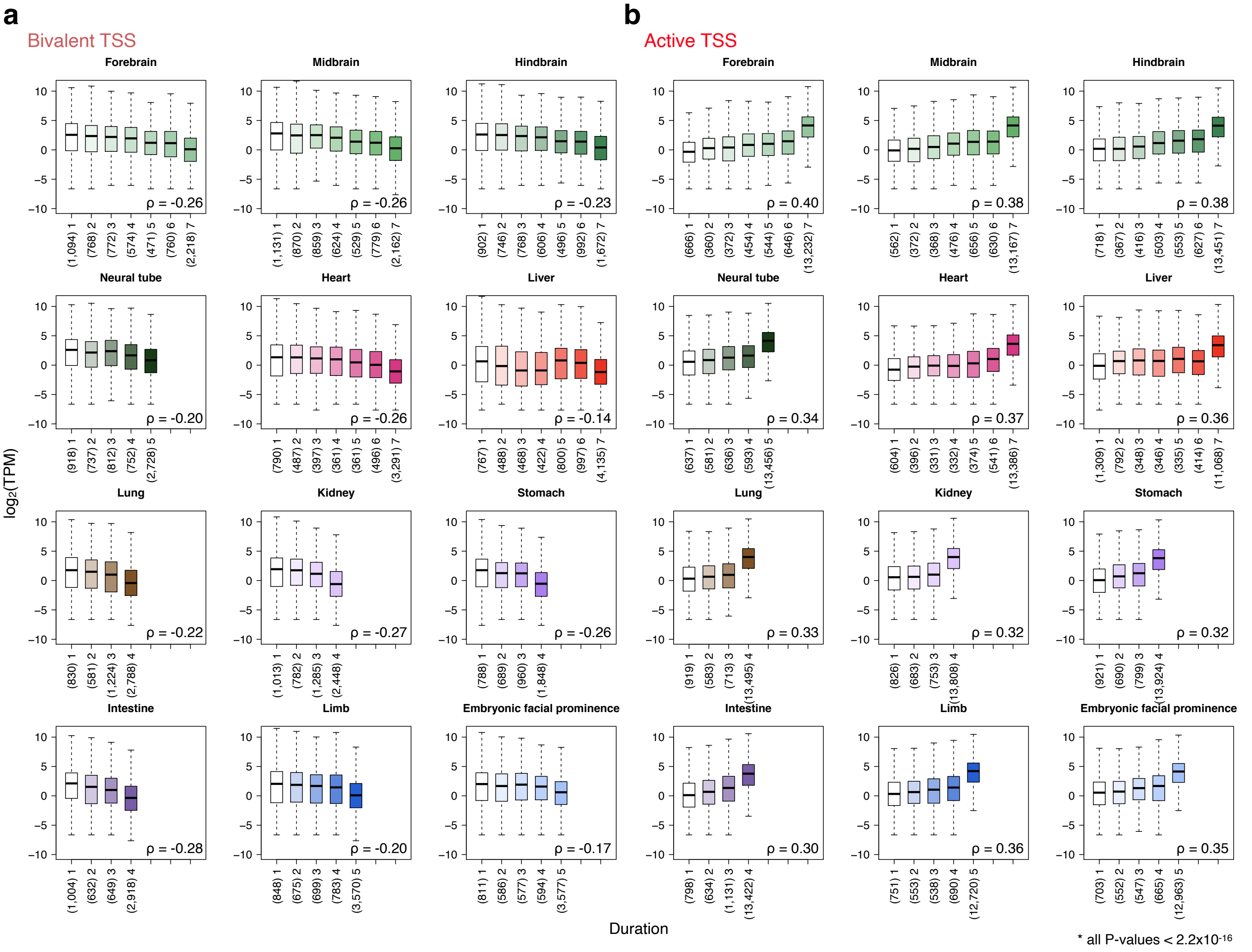

Supplementary Fig. 8: Correlations between gene expression and the duration of bivalent and active TSSs.
The expression levels of genes with certain duration (number of time-points) of being bivalent (**a**) or active (**b**) in each tissue. Numbers in parentheses indicate the number of genes for each duration. P-values were computed with ANOVA with multiple-testing correction. For all box-plots, whiskers show 95% confidence intervals, boxes represent the first and third quartiles, the vertical midline is the median, and outliers are omitted.

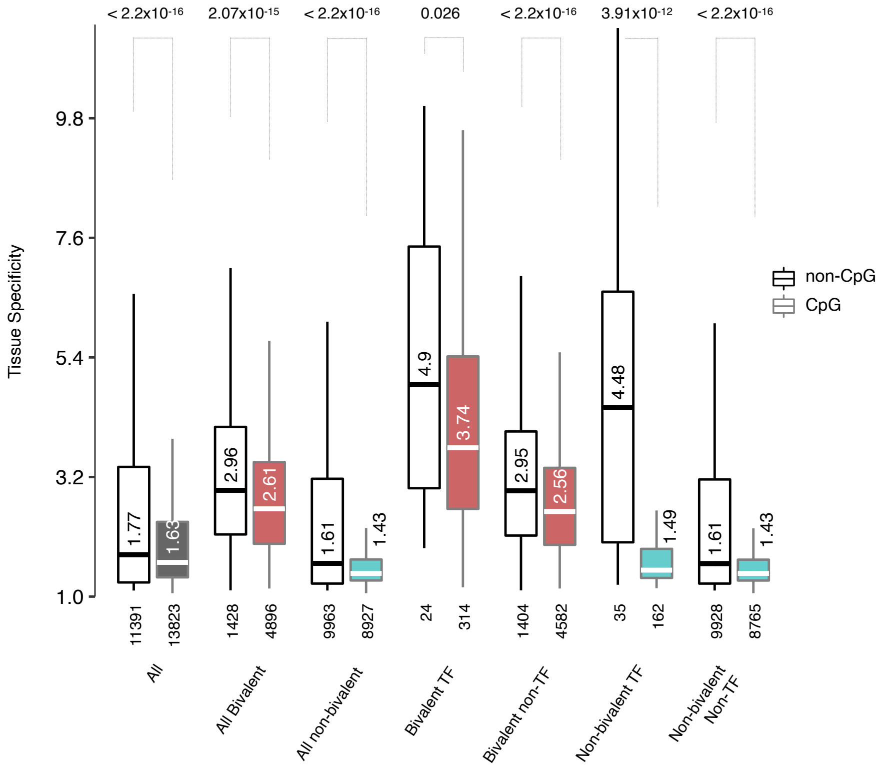

Supplementary Fig. 9: Tissue specificity for groups of genes classified by whether their TSSs overlap CpG islands. Tissue-specificity scores are shown for all genes, and subsets of genes depending on whether they encoded TFs, they have a bivalent TSS, and whether the TSSs overlap CpG islands. Two-sided Wilcoxon P-values for comparing the CpG and non-CpG groups of genes are provided. For all box-plots, whiskers show 95% confidence intervals, boxes represent the first and third quartiles, the vertical midline is the median, and outliers are omitted.

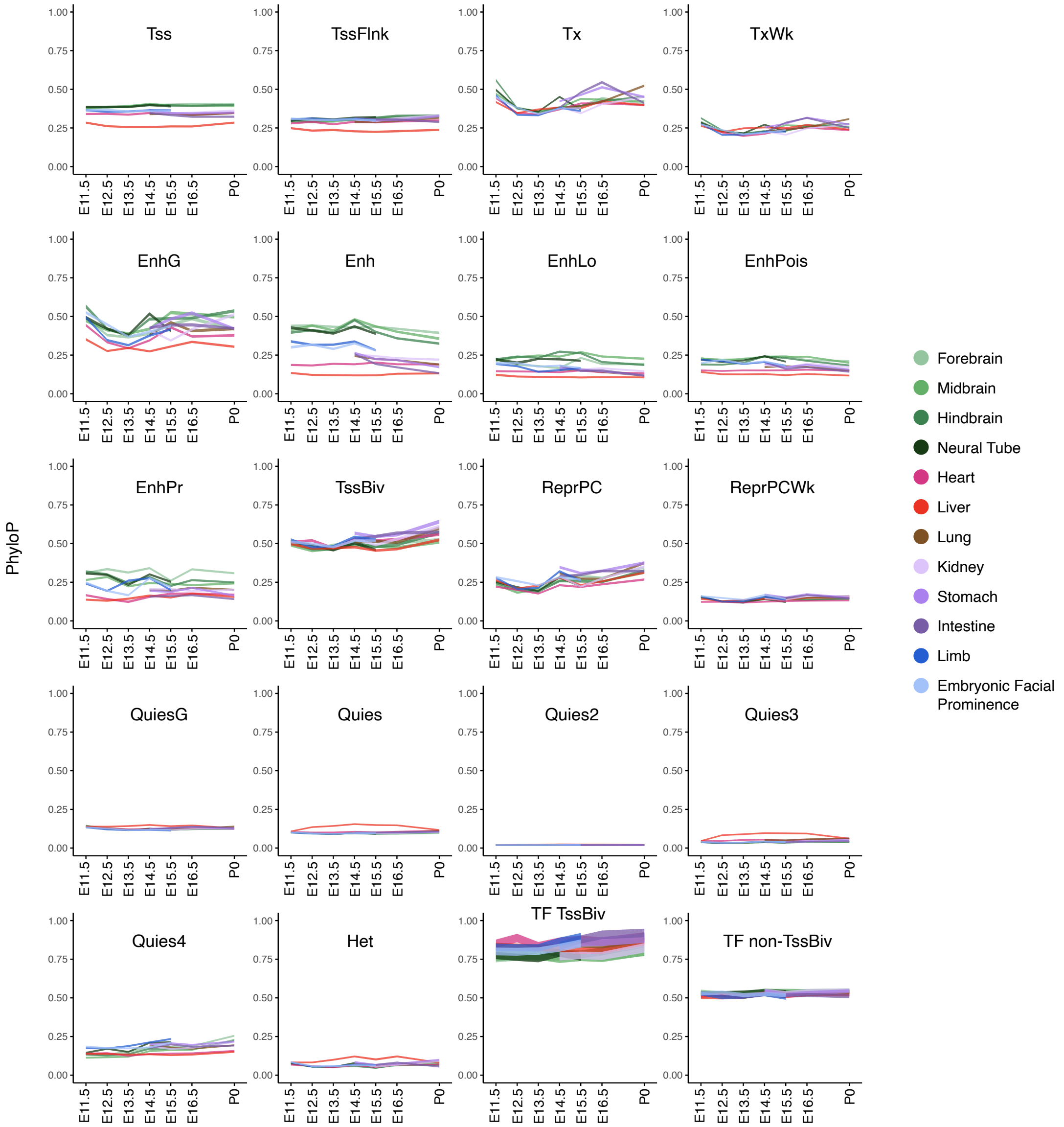

Supplementary Fig. 10: Evolutionary conservation for genomic bins assigned to each chromatin state in each biosample. Average PhyloP scores are plotted for the 18 chromatin states. The last two panels (bottom right) are TSSs of transcription factors stratified by whether they fall in a TssBiv genomic bin or not. The thickness of a line corresponds to the standard error. Tissues are colored accordingly.

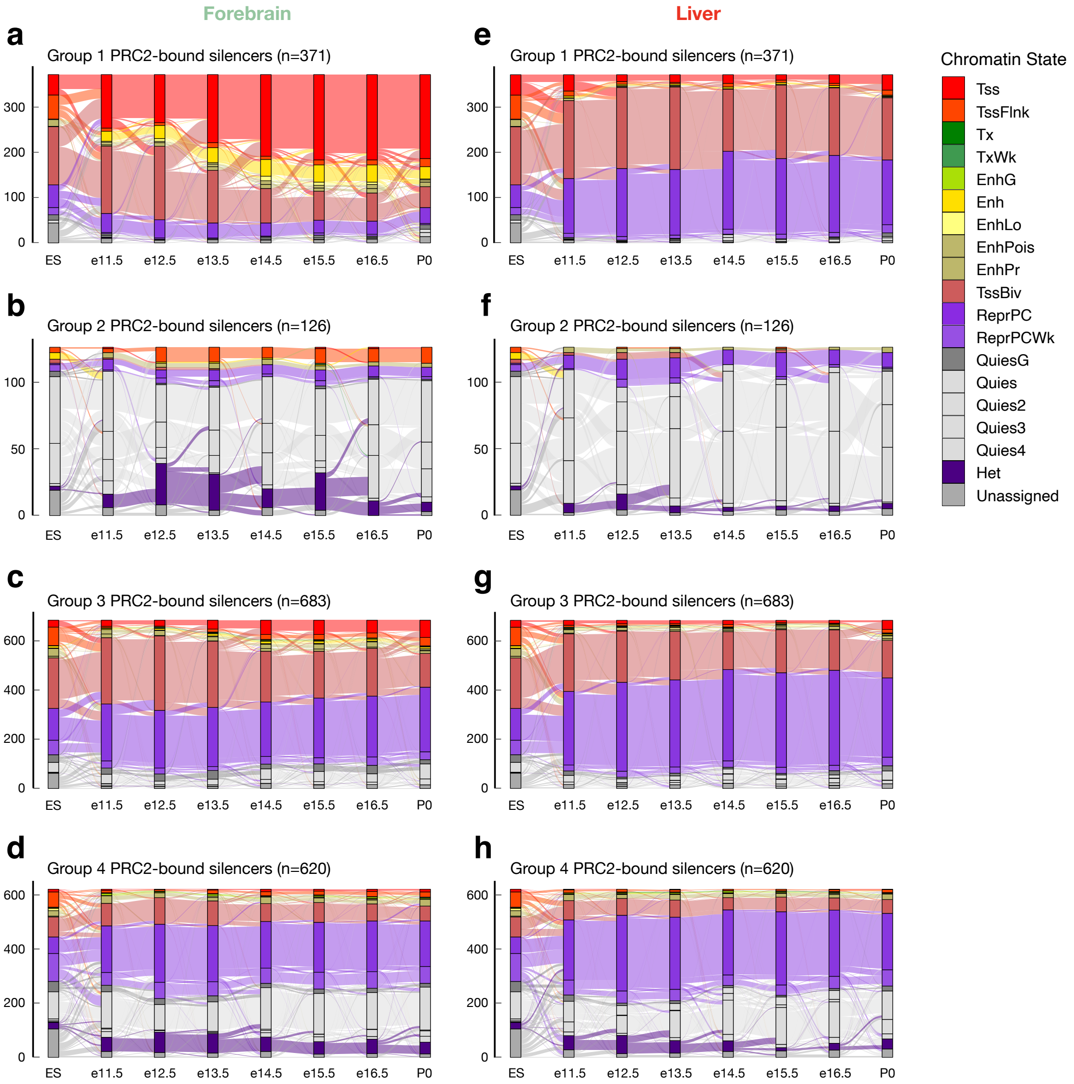

Supplementary Fig. 11: Chromatin state assignments for the center positions of PRC2-bound silencers.
Four groups of PRC2-bound silencers correspond to those in Fig. 6a are plotted across time-points in the forebrain (**a-d**) and liver (**e-h**). The state assignments for mouse embryonic stem cells (ES) are included for comparison. States are colored as in Fig. 1c.

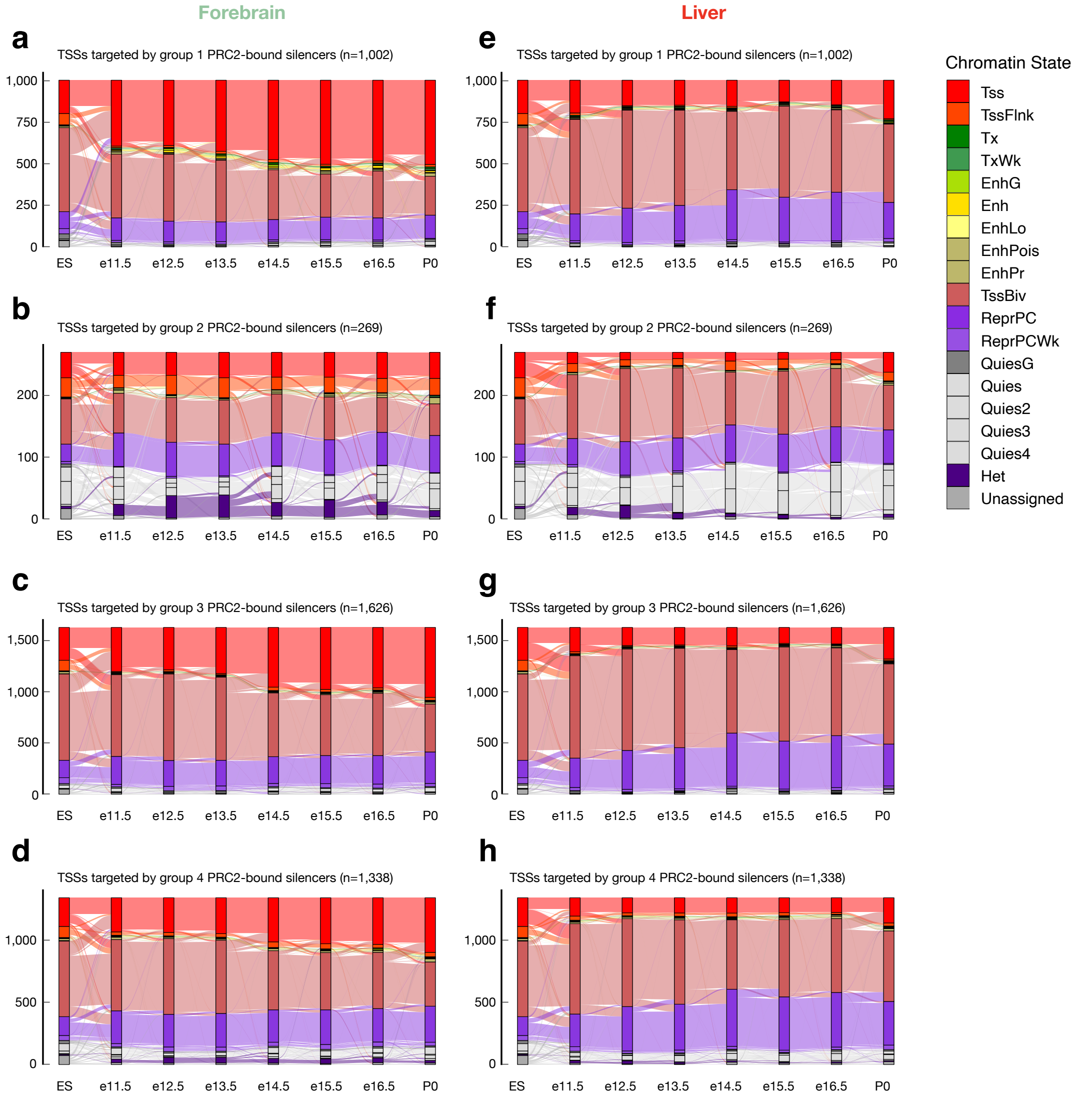

Supplementary Fig. 12: Chromatin state assignments for the TSSs targeted by PRC2-bound silencers. This figure corresponds to supplementary Fig. 11 but for the TSSs targeted by PRC2-bound silencers.
