# Supplementary material for: Annotation of chromatin states in 66 complete mouse epigenomes during development
Source: Commun Biol. 2021 Feb 22;4:239. doi: 10.1038/s42003-021-01756-4 (PMC7900196; doi:10.1038/s42003-021-01756-4)
Supplement: Supplementary file 2 — Description of Additional Supplementary Files [file 42003_2021_1756_MOESM2_ESM.pdf]

Description of Additional Supplementary Files

File Name: Supplementary Data 1

Description: Input datasets and their ENCODE accessions. ENCODE file accession IDs for all input files. a. BAM files for histone ChIP-seq datasets and controls. b. BED files with CpG calls from WGBS. c. RNA-seq TPM matrices for the two replicates of each biosample. d. BAM files for ATAC-seq. e. BAM files for DNase-seq.

File Name: Supplementary Data 2

Description: Bivalent TSSs in each biosample. GENCODE M4 TSS annotations were intersected with bivalent regions in each biosample. Sites occupying the same genomic position were merged.

File Name: Supplementary Data 3

Description: Bivalent genes and their expression levels. a. Expression levels are reported in TPM, in each tissue and time-point. b. The number of bivalent genes shared between any pair of biosamples. c. The number of bivalent genes shared between any pair of tissues. Diagonal numbers indicate the total number of bivalent genes in each tissue. d. Bivalent state of the TSSs of the genes in each biosample. e. Bivalent regions defined across all biosamples. f. Union of bivalent regions detected in all biosamples, as determined by regular expression (see Methods).

File Name: Supplementary Data 4

Description: GO enrichment analysis using the PANTHER tool. a. PANTHER output for genes that are bivalent in all tissues. b. List of genes submitted for analysis in a. c. PANTHER output for genes that are bivalent exclusively in the liver. d. List of genes submitted for analysis in c.
