# Supplementary material for: Annotation of chromatin states in 66 complete mouse epigenomes during development
Source: Commun Biol. 2021 Feb 22;4:239. doi: 10.1038/s42003-021-01756-4 (PMC7900196; doi:10.1038/s42003-021-01756-4)
Supplement: Supplementary file 7 — Reporting Summary [file 42003_2021_1756_MOESM7_ESM.pdf]

nature research

Corresponding author(s): Zhiping Weng

Last updated by author(s): Jan 12, 2021

Reporting Summary

Nature Research wishes to improve the reproducibility of the work that we publish. This form provides structure for consistency and transparency in reporting. For further information on Nature Research policies, see our Editorial Policies and the Editorial Policy Checklist.

Statistics

For all statistical analyses, confirm that the following items are present in the figure legend, table legend, main text, or Methods section.

n/a	Confirmed	
☐	☒	The exact sample size (n) for each experimental group/condition, given as a discrete number and unit of measurement
☐	☒	A statement on whether measurements were taken from distinct samples or whether the same sample was measured repeatedly
☐	☒	The statistical test(s) used AND whether they are one- or two-sided<br>*Only common tests should be described solely by name; describe more complex techniques in the Methods section.*
☐	☒	A description of all covariates tested
☐	☒	A description of any assumptions or corrections, such as tests of normality and adjustment for multiple comparisons
☐	☒	A full description of the statistical parameters including central tendency (e.g. means) or other basic estimates (e.g. regression coefficient) AND variation (e.g. standard deviation) or associated estimates of uncertainty (e.g. confidence intervals)
☐	☒	For null hypothesis testing, the test statistic (e.g. F, t, r) with confidence intervals, effect sizes, degrees of freedom and P value noted<br>*Give P values as exact values whenever suitable.*
☐	☒	For Bayesian analysis, information on the choice of priors and Markov chain Monte Carlo settings
☐	☒	For hierarchical and complex designs, identification of the appropriate level for tests and full reporting of outcomes
☐	☒	Estimates of effect sizes (e.g. Cohen's d, Pearson's r), indicating how they were calculated

Our web collection on statistics for biologists contains articles on many of the points above.

Software and code

Policy information about availability of computer code

Data collection	All experimental data used in this paper can be accessed at the encode Portal (http://www.encodeproject.org/), using the accession IDs listed in Supplementary Data 1.
Data analysis	The code used to extract genomic regions based on regular expression can be found on GitHub, at https://github.com/weng-lab/stateregexp.git.<br>ChromHMM: http://compbio.mit.edu/ChromHMM/<br>bedtools: https://bedtools.readthedocs.io/en/latest/<br>USCS Kent tools: https://github.com/ENCODE-DCC/kentUtils<br>UMAP: https://cran.r-project.org/web/packages/umap/vignettes/umap.html<br>PANTHER: http://pantherdb.org/<br>deeptools: https://deeptools.readthedocs.io/en/develop/

For manuscripts utilizing custom algorithms or software that are central to the research but not yet described in published literature, software must be made available to editors and reviewers. We strongly encourage code deposition in a community repository (e.g. GitHub). See the Nature Research guidelines for submitting code & software for further information.

Data

Policy information about availability of data

All manuscripts must include a data availability statement. This statement should provide the following information, where applicable:

- Accession codes, unique identifiers, or web links for publicly available datasets
- A list of figures that have associated raw data
- A description of any restrictions on data availability

All experimental data used in this paper can be accessed at the encode Portal (http://www.encodeproject.org/), using the accession IDs listed in Supplementary Table 1. The chromatin state assignments for both the eight-mark, 15-state model and the ten-mark, 18-state model are also accessible at the ENCODE Portal (https://www.encodeproject.org/search/?searchTerm=ChromHMM+Zhiping+Weng&type=FileSet). State annotations with posterior probabilities for all 200bp genomic windows can be found at https://doi.org/10.6084/m9.figshare.13271705. Per-window PhyloP scores and state durations are available at https://doi.org/10.6084/m9.figshare.13271684. Epigenetic signals of Enh states and TssBiv states for UMAP analysis are available at https://doi.org/10.6084/m9.figshare.13271717.

We made a track hub (https://users.wenglab.org/vanderva/trackhub/chromhmmpaper/hub_0.txt) for the UCSC genome browser73 to visualize all the data and annotations used in this study listed below. The trackhub can be accessed via a UCSC session: https://genome.ucsc.edu/s/Kaili/ChromHMM_paper.

Field-specific reporting

Please select the one below that is the best fit for your research. If you are not sure, read the appropriate sections before making your selection.

☒ Life sciences ☐ Behavioural & social sciences ☐ Ecological, evolutionary & environmental sciences

For a reference copy of the document with all sections, see nature.com/documents/nr-reporting-summary-flat.pdf

Life sciences study design

All studies must disclose on these points even when the disclosure is negative.

Sample size	No experiments were performed.
Data exclusions	No data was excluded
Replication	No experiments were performed.
Randomization	No experiments were performed.
Blinding	No experiments were performed.

Reporting for specific materials, systems and methods

We require information from authors about some types of materials, experimental systems and methods used in many studies. Here, indicate whether each material, system or method listed is relevant to your study. If you are not sure if a list item applies to your research, read the appropriate section before selecting a response.

Materials & experimental systems

n/a	Involved in the study	
☒	☐	Antibodies
☒	☐	Eukaryotic cell lines
☒	☐	Palaeontology and archaeology
☒	☐	Animals and other organisms
☒	☐	Human research participants
☒	☐	Clinical data
☒	☐	Dual use research of concern

Methods

n/a	Involved in the study	
☒	☐	ChIP-seq
☒	☐	Flow cytometry
☒	☐	MRI-based neuroimaging
